# Induction of kidney-related gene programs through co-option of *SALL1* in mole ovotestes

**DOI:** 10.1242/dev.201562

**Published:** 2023-09-05

**Authors:** Magdalena Schindler, Marco Osterwalder, Izabela Harabula, Lars Wittler, Athanasia C. Tzika, Dina K. N. Dechmann, Martin Vingron, Axel Visel, Stefan A. Haas, Francisca M. Real

**Affiliations:** ^1^ Gene Regulation & Evolution, Max Planck Institute for Molecular Genetics, Berlin 14195, Germany; ^2^ Institute for Medical and Human Genetics, Charité - Universitätsmedizin Berlin, Berlin 13353, Germany; ^3^ Department for BioMedical Research (DBMR), University of Bern, Bern 3008, Switzerland; ^4^ Department of Cardiology, Bern University Hospital, Bern 3010, Switzerland; ^5^ Environmental Genomics and Systems Biology Division, Lawrence Berkeley National Laboratory, 1 Cyclotron Road, Berkeley, CA 94720, USA; ^6^ Epigenetic Regulation and Chromatin Architecture, Max-Delbrück-Centrum für Molekulare Medizin (MDC), Berlin 10115, Germany; ^7^ Department of Developmental Genetics, Transgenic Unit, Max Planck Institute for Molecular Genetics, Berlin 14195, Germany; ^8^ Department of Genetics & Evolution, University of Geneva, Geneva 1205, Switzerland; ^9^ Department of Migration, Max Planck Institute for Animal Behavior, Radolfzell 78315, Germany; ^10^ Department of Biology, University of Konstanz, Konstanz 78457, Germany; ^11^ Department of Computational Molecular Biology, Max Planck Institute for Molecular Genetics, Berlin 14195, Germany; ^12^ Department of Energy Joint Genome Institute, Berkeley, CA 94720, USA; ^13^ School of Natural Sciences, University of California, Merced, CA 95343, USA

**Keywords:** Evolutionary genomics, Gene regulation, Gonad development, Moles, Ovotestes, SALL1

## Abstract

Changes in gene expression represent an important source of phenotypic innovation. Yet how such changes emerge and impact the evolution of traits remains elusive. Here, we explore the molecular mechanisms associated with the development of masculinizing ovotestes in female moles. By performing integrative analyses of epigenetic and transcriptional data in mole and mouse, we identified the co-option of *SALL1* expression in mole ovotestes formation. Chromosome conformation capture analyses highlight a striking conservation of the 3D organization at the *SALL1* locus, but an evolutionary divergence of enhancer activity. Interspecies reporter assays support the capability of mole-specific enhancers to activate transcription in urogenital tissues. Through overexpression experiments in transgenic mice, we further demonstrate the capability of *SALL1* to induce kidney-related gene programs, which are a signature of mole ovotestes. Our results highlight the co-option of gene expression, through changes in enhancer activity, as a plausible mechanism for the evolution of traits.

## INTRODUCTION

Coordinated gene expression represents the cornerstone of developmental processes and homeostasis. In animals, transcription is controlled mainly by the action of cis-regulatory elements (CREs), such as enhancers, which control gene expression patterns with spatial and temporal precision. CREs control tissue-specific aspects of gene expression, acting in cooperation to constitute complex and pleiotropic gene expression patterns (Long et al., 2016). To exert their function, CREs enter into physical proximity with gene promoters, mediated by the 3D folding of chromatin. CRE-promoter interactions are framed within topologically associating domains (TADs), which are 3D chromatin structures containing loci that interact with increased frequencies and are shielded from the regulatory influence of other genomic regions (Nora et al., 2012; Dixon et al., 2012).

Coding mutations generally alter all the different functions of a gene, thus inducing systemic effects that might be detrimental to the development of an organism. In contrast, mutations in CREs display tissue-specific effects, thus preserving essential gene functions in other tissues. Consistently, the multiplicity of CREs can confer variations in expression patterns that contribute to gene pleiotropy, and support the rapid evolvability of these non-coding elements (Wray, 2007). Indeed, mutations altering regulatory elements have been associated with the emergence of certain traits, such as the evolution of limbs in ungulate animals (Lopez-Rios et al., 2014).

Furthermore, the repurposing of a gene or regulatory element to a new function through a co-option process also represents an important source of phenotypic innovation (Sanetra et al., 2005; McLennan, 2008; Holland, 2013). This mechanism has been exemplified in the evolution of the neural crest cells in vertebrates through the acquisition of new regulatory elements for the *SoxE* family genes (Jandzik et al., 2015). Another relevant example of co-option is illustrated by the mechanism through which the propagation of retroviruses in the mammalian genomes has shaped the regulatory landscape of the immune system (Chuong et al., 2016). Therefore, variations in gene expression and function, through CRE mutations, underlie the evolution of certain phenotypic traits and can represent the basis for species adaptation.

A prominent example of phenotypic evolution is observed in Talpid moles. Unlike most mammalian species, female moles consistently develop ovotestes instead of ovaries. These gonads are composed of ovarian tissue, supporting a fertile function, and a sterile testicular region that secretes male hormones. These hormones exert a masculinizing effect in female moles, increasing muscle strength and aggression, aspects that likely contribute to their adaptation to subterranean environments. In a previous study, we demonstrated that the evolution of ovotestes is associated with the reorganization of TADs, which alter CRE-promoter interactions and gene expression patterns (Real et al., 2020). In particular, a large inversion relocates active enhancers in the vicinity of the pro-testicular gene *FGF9*, the ectopic expression of which in female gonads leads to meiosis inhibition and masculinization. In addition, a duplication of enhancer elements is associated with the increased expression of *CYP17A1*, which encodes an enzyme for male hormone synthesis and increased muscle strength. Although the observed regulatory changes at these loci partially explain the mole phenotype, it is plausible that additional mechanisms contribute to the evolution of this trait.

In this study, we have further investigated the molecular mechanisms associated with mole ovotestis development. Using integrative epigenetic and transcriptional approaches in mole and mouse, we identify that the expression of the transcription factor *SALL1* has been co-opted in the formation of XX testicular tissue in the Iberian mole *Talpa occidentalis*, through changes in CREs. Our finding is further supported by expression analyses in closely related species that develop normal ovaries, such as shrews and hedgehogs. We determine the regulatory landscape of this gene, highlighting an evolutionary conserved TAD structure, but with divergent enhancer activity. Through *in vivo* interspecies reporter assays, we reveal the potential of enhancer elements to evolve new activity domains in moles. By using transgenic mice that overexpress *Sall1* in ovaries, we demonstrate the capacity of this factor to activate kidney-related gene programs that are also observed during mole ovotestis formation. Altogether, our results further extend our understanding of the molecular basis of a unique trait, highlighting the important role of regulatory variation in evolution.

## RESULTS

### Evolutionary conservation of mammalian gonadal enhancers

**Fig. 1.**
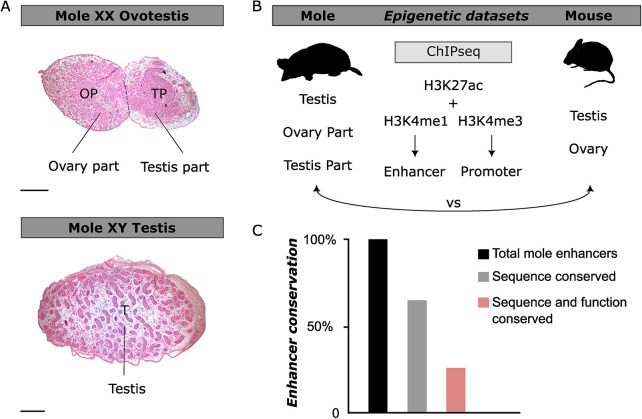
**Characterization of regulatory elements in mole ovotestes.** (A) Hematoxylin and Eosin staining of mole gonads at postnatal stage P7. Female ovotestis in upper panel; testis in lower panel. OP, ovary part; TP, testis part; T, testis. There is clear separation of the ovotestis into two parts. Scale bars: 100 µm. (B) Schematic of the gonadal tissues sampled to generate the epigenetic datasets in mole and mouse. Five tissues and three different histone modifications were used for the ChIP-seq experiments. vs, versus. (C) Percentage of mole enhancers conserved compared with mice. Conservation at the sequence level is shown in gray, conservation at the enhancer signature level is shown in light red.

CREs represent a major source of tissue-specific gene expression (Long et al., 2016). We previously explored the regulatory landscape of mole developing gonads, at an early postnatal stage (7 days post-partum – stage P7) (Real et al., 2020). At this developmental time-point, testicular and ovarian tissues from female ovotestes are first morphologically discernable and can be microdissected (Fig. 1A). Furthermore, Leydig cells of the testicular part differentiate and produce testosterone, whereas meiosis initiates in the ovarian part, an event considered to be one of the earliest signs of female gonadogenesis in mammals (McLaren, 2003). We identified regulatory elements in mole gonads by performing ChIP-seq experiments against a combination of histone marks, H3K27ac together with H3K4me1 and H3K4me3, for the distinction of enhancers and promoters, respectively. By using the tool CRUP (Ramisch et al., 2019), we combined these datasets in each sampled tissue to call and rank active regulatory regions according to their enhancer probability score (Fig. 1B).

To explore the degree of conservation of the enhancer landscape in moles, we generated analogous datasets from mouse gonads, at a time point when Leydig cells differentiate, and meiosis takes place (E13.5; Fig. 1B). By comparing mole and mouse gonadal epigenetic datasets, we observed that from the 70,618 predicted enhancers in mole gonads ∼65% are conserved to some extent at the sequence level, meaning they can be lifted over to the mouse genome (Fig. 1C). However, only 25% of those enhancers are active in both species, meaning that they share an active enhancer signature in both mole and mouse gonads. Accordingly, ∼40% of the predicted sequence conserved enhancers represent mole-specific regulatory regions and are thus potentially associated with characteristics of this species. Therefore, our results imply a repurposing of enhancer function during gonad evolution.

### Co-option of SALL1 expression in mole ovotestis formation

**Fig. 2.**
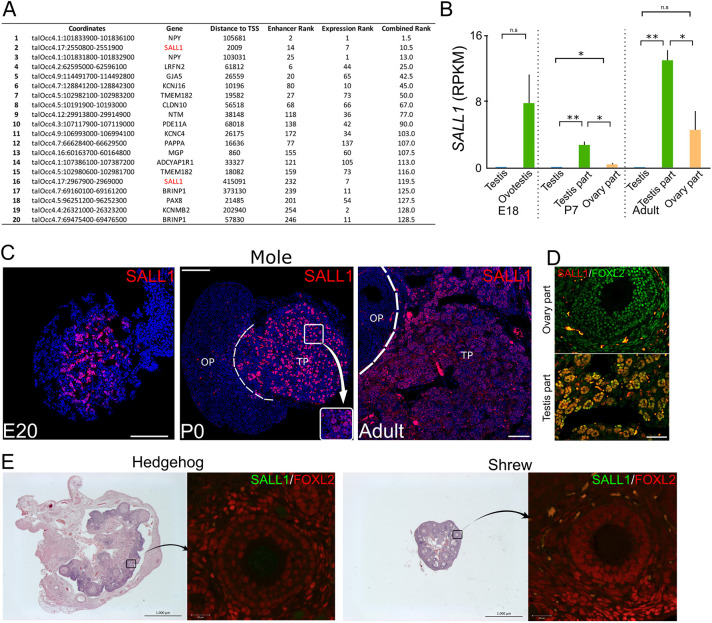
**Identification of *SALL1* as a marker for testis part formation in mole ovotestes.** (A) Top 20 enhancer regions ranked by enhancer score and specificity of expression of the associated gene in the testis part of the ovotestis. Two *SALL1* enhancers are highly ranked (2 and 16). (B) *SALL1* expression levels in RPKM (reads per kilobase million) from mole RNA-seq data at different developmental time points. (C) Spatio-temporal profile of SALL1 expression in mole ovotestes (immunofluorescence; SALL1 in red, DAPI in blue). SALL1 is spatially restricted to the medullary (testicular) region of the mole ovotestis at E20 and is also present in the testis part thereafter. Inset shows localization to Sertoli-like cells. OP, ovarian part; TP, testicular part. Scale bars: 100 µm. (D) Double immunostaining for SALL1 and FOXL2 in adult ovotestes. SALL1-positive cells are absent in the ovary part, contrary to the testis part, where both markers colocalize in the spherules (equivalent to testis cords). Scale bar: 100 µm. (E) Spatial expression of SALL1 is absent in adult female hedgehog (*Atelerix albiventris*, left) and adult female shrew (*Sorex araneus*, right) (immunofluorescence; SALL1 in green, ovarian marker FOXL2 in red). Scale bars: 1000 µm (black); 20 µm (white).

Our approach identified a subset of 6419 mole-specific enhancers that are only active in the testicular part of the ovotestis and could potentially contribute to the development of this unique tissue. We then explored whether these enhancers are associated with the acquisition of specific transcriptional signatures using RNA-seq datasets from the same developmental stage. We therefore jointly ranked enhancers by specificity in enhancer probability in the testicular part of the ovotestes and by the specific expression of their putative target gene in the same tissue. We defined the putative target genes of each enhancer as the gene with the closest transcription start site to the enhancer region within the same TAD. This approach prioritizes genes whose respective regulatory domain contains enhancer elements specifically active in the testicular part compared with the ovary part and the male testis (Fig. 2A, Table S1). The top-ranking genes identified by this approach were *NPY* and *SALL1. NPY* is a hormone neuropeptide expressed in Leydig cells (Adrian et al., 1983; Körner et al., 2011), whereas *SALL1* is a transcription regulator involved in cell fate decision (Sweetman and Münsterberg, 2006). *SALL1* is usually expressed during development in embryonic tissues, including eye, neural tube, limb or kidney (Nishinakamura and Takasato, 2005). Strikingly, our RNA-seq data revealed that *SALL1* is highly expressed in the testicular part of mole ovotestes at P7, but not in the XY testis or the XX ovarian region. In fact, *SALL1* is highly expressed already in the early embryonic ovotestis and becomes specific to the testis part as the organ differentiates (Fig. 2B). In humans, mutations in *SALL1* are associated with a congenital malformation syndrome that affects limbs, kidneys and ears (Townes Brocks syndrome, OMIM 107480) (Kohlhase et al., 1998). *SALL1* misexpression has also been linked to certain types of androgen-producing ovarian tumors (Ma et al., 2002), indicating that it might be involved in re-programming ovarian cells.

To further explore the spatio-temporal dynamics of *SALL1* expression, we performed immunostaining in mole gonads at different stages of development (Fig. 2C). This analysis revealed that SALL1 expression is specific to the mole female gonad and, importantly, that this expression is spatially restricted to the medullary region of the developing ovotestis, which is the precursor of the testicular tissue. Double immunostaining for SALL1 and FOXL2, a marker of female somatic cells (Nicol et al., 2018) (Fig. 2D) confirmed that SALL1 expression is restricted to the testicular part of the ovotestis. This staining also revealed that cells that are simultaneously positive for FOXL2 and SALL1 form the spherules, which are equivalent to testis cords and considered ‘Sertoli-like’ cells. Based on these results, the expression of SALL1 detected in the RNA-seq from the ovarian part of the adult ovotestis (Fig. 2B) is likely due to imperfect dissection of the tissue, which is especially challenging, as the two gonadal compartments are intricately connected at this stage. Therefore, the expression pattern of *SALL1* is constant during the entire development and persists in adulthood, thus constituting a bona-fide marker for the testicular tissue of mole ovotestis.

We then explored the evolutionary conservation of *SALL1* expression in other mammalian species. We examined the pattern of expression of *Sall1* in mice by immunostaining and transcriptomic analyses. Immunostaining analyses showed a complete absence of SALL1 protein in mouse gonads at embryonic stage E13.5; however, the protein could be detected in known *Sall1*-expressing tissues, such as the embryonic kidneys (Fig. S1A). This observation is extended to adulthood, where RNA-seq data shows practically no expression in both males and females when compared with the mole (Fig. S1B). We further expanded our analysis of SALL1 expression to also include species from the order *Eulipotyphla*, which are evolutionarily close to moles (Douady et al., 2002). Specifically, we analyzed ovarian samples from the hedgehog *Atelerix albiventris*, as well as from the common shrew, *Sorex araneus*, the latter species belonging to the closest taxonomic group but developing normal ovaries. Immunostaining analyses showed the absence of SALL1 expression in the gonads of these two species (Fig. 2E). However, we could detect SALL1 in other control tissues such as neural tube or kidney from hedgehog and shrew, proving the specificity of the antibody used (Fig. S2A,B). In addition, the absence of expression of SALL1 in the ovaries of these species was further confirmed by RT-qPCR (Fig. S2C,D). Overall, these results indicate that *SALL1* expression has been acquired during the evolution of mole ovotestes.

### Conserved 3D organization but divergent enhancers at the mole SALL1 locus

**Fig. 3.**
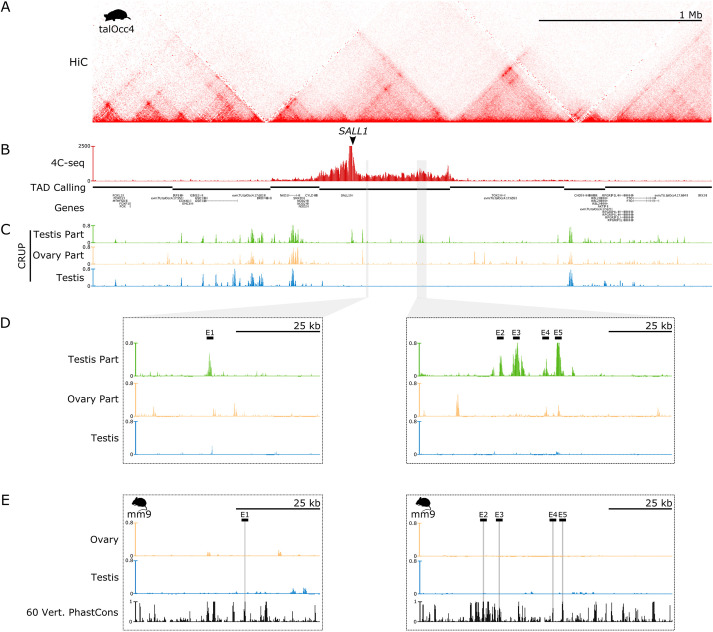
**Regulatory domains and the epigenetic landscape of *SALL1*.** (A) Hi-C map from mole embryonic limbs denotes the domain of *SALL1* in a large gene desert. (B) 4C-seq analysis from the female adult testis part with *SALL1* promoter as a viewpoint. There is a high interaction frequency between the gene promoter and the surrounding 1 Mb desert that clearly demarcates the *SALL1* regulatory domain. (C) Epigenetic landscape of *SALL1* in the three tissues sampled with the tool CRUP. Numerous active enhancers are present in the testicular part of the ovotestis where *SALL1* is specifically expressed. (D) Zoom in on two mole regions containing five specific regulatory elements for the testis part of the ovotestes, named as enhancers 1 to 5 (E1-5). (E) Regions homologous to the testis part enhancers in the mouse genome (gray bars). Enhancer activity is absent in these regions.

To define the regulatory landscape of *SALL1*, we examined previously published Hi-C data from different mole tissues (Real et al., 2020) (Fig. 3A, Fig. S3). Chromatin interaction maps revealed a large 1 Mb TAD, in which *SALL1* is the only protein-coding gene. The interaction profile of *SALL1* in the testicular part of the ovotestis was further explored at increased resolution through circular chromosome conformation capture (4C-seq), using the gene promoter as a viewpoint (Fig. 3B). These experiments demonstrate prominent interactions of *SALL1* across the entire TAD, with a sharp decrease in contacts outside this domain. We then explored the degree of conservation of the *SALL1* interaction profile by comparing the mole against mouse data (Bonev and Cavalli, 2016). This comparison revealed that, despite notable differences in *SALL1* expression, the locus displays a remarkable preservation of its 3D structure across species (Fig. S4A).

Next, we overlaid the *SALL1* interaction profile to the epigenetic datasets, to identify potential regulatory elements (Fig. 3C). This revealed several candidate enhancer regions that were active exclusively in the testicular part of the ovotestis. Specifically, we identified one putative enhancer element that is close to *SALL1* and unique for the testicular region, as well as a distant cluster of four additional elements. This putative enhancer cluster is indeed in close physical proximity to the *SALL1* promoter, as denoted by a specific loop in the Hi-C map and an increase in contacts in the 4C profile. A zoom-in on these regions highlights the specificity of these enhancers for the testicular part of the ovotestes (Fig. 3D). Consistent with its conserved 3D structure, these candidate enhancers lie in syntenic regions when aligned against mouse or shrew genomes (Fig. S5). However, a comparison with the respective mouse epigenetic datasets revealed that these elements were not active in mouse gonads (Fig. 3E). Specific alignments of these five enhancers against mouse and shrew revealed only a partial degree of sequence conservation (Figs S6 and S7).

**Fig. 4.**
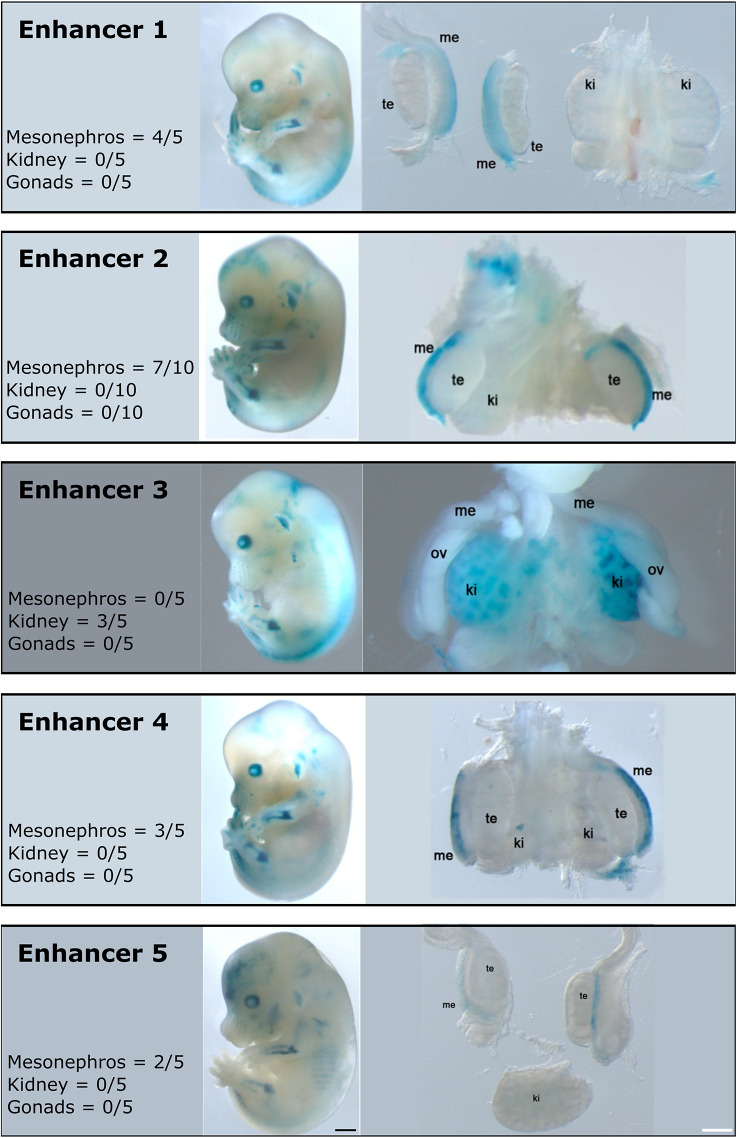
***lacZ* reporter assays for enhancer elements E1-5 associated with *SALL1*.** The enhancer activity of each element is depicted in separate boxes 1 to 5. Entire embryos at E13.5, as well as the dissected urogenital tracts are displayed. Me, mesonephros; te, testes; ov, ovaries; ki, kidneys. Scale bars: 1000 µm (black); 100 µm (white).

To validate the activity of these putative enhancers *in vivo*, we tested the five mole regions for enhancer activity in mouse transgenic *lacZ* reporter assays (Visel et al., 2007) (E1-E5; Fig. 3D). Of note, these elements display active enhancer marks that are specific for the testis part of the ovotestes, but such marks are not present in mouse gonadal tissue. At E13.5, all regions tested showed reproducible tissue-restricted activity, thus confirming them as true enhancers (Fig. 4; Figs S8-S12). Enhancer activity was observed in several tissues, such as the limbs or eyes, in which *Sall1* is known to be expressed. Interestingly, enhancer 3 displayed specific activity in kidneys, another *Sall1*-expressing tissue (Nishinakamura and Takasato, 2005), which is consistent with its predicted enhancer activity in mouse embryonic kidneys (Fig. S4B). Although none of these enhancers induced reporter expression in developing gonads, enhancers 1, 2, 4 and 5 were active in the adjacent mesonephros. This tissue has the same ontogenetic origin as the gonads, and contributes to its cellular composition through cell migration (Tilmann and Capel, 1999). Furthermore, it has been previously shown that SALL1 is expressed in the mesonephric duct of mice (Nishinakamura et al., 2001), a pattern that is also conserved in moles (Fig. S13).

We sought to investigate whether the divergence observed in the mole enhancer sequences included alterations in transcription factor binding sites that may explain the specific activation of *SALL1* in the ovotestis. To this end, we conducted a transcription factor enrichment analysis on the five mole enhancer sequences and compared them with mouse and shrew sequences. We further filtered putative motifs for transcription factors expressed in mole gonadal tissues. The results revealed a distinctive binding pattern among species (Table S2), with minimal overlap in the most significant transcription factor bindings (Fig. S14A). Moreover, we observed higher expression of some top-ranked transcription factors, such as *IRF4* or *FOXP1*, in the testicular part of the ovotestes compared with mice (Fig. S14B), which could account for the lack of *lacZ* activity in mouse gonads. This observation, together with the moderate sequence conservation compared with other mammals suggests that the evolution of enhancers in the regulatory domain of *SALL1* may have driven its expression in the testicular part of mole ovotestis.

### SALL1 expression triggers kidney-related gene programs during ovarian development

**Fig. 5.**
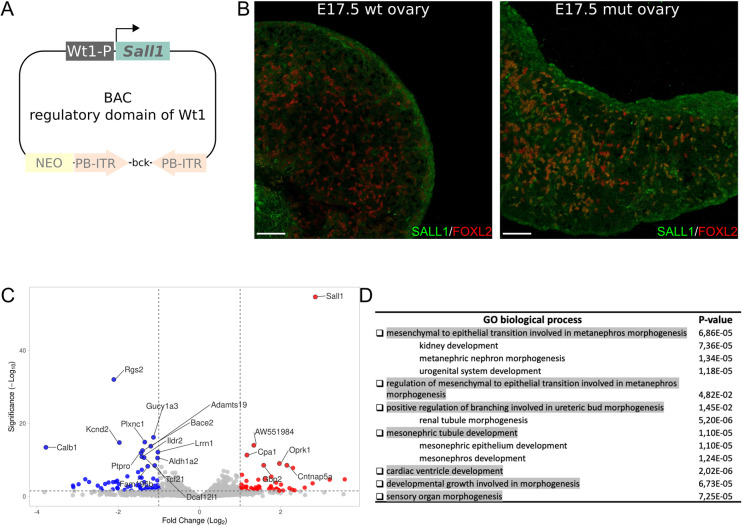
**Overexpression of *Sall1* in mouse embryonic ovaries results in hundreds of deregulated genes.** (A) Cloning strategy to overexpress *Sall1* in somatic ovarian cells through BAC transgenesis. *Sall1* is regulated under the promoter and regulatory regions of the gonadal somatic gene *Wt1*. (B) Immunostaining against SALL1 (green) and FOXL2 (red) in wild-type and mutant ovaries at E17.5. There is a high abundance of SALL1 and FOXL2 double-positive cells (orange) in the mutant gonad, confirming the overexpression success. Scale bars: 100 µm. (C) Volcano plot from RNA-seq of mutant ovaries compared with control ovaries from littermates at E13.5. The 20 most deregulated genes are indicated. *Sall1* is the most significantly upregulated gene, as shown in the upper right corner of the plot. The *x*-axis shows the expression changes in Log2 fold-change and the *y*-axis shows the *P*-value. (D) Gene ontology enrichment analyses of the common upregulated genes in the *Sall1* mutant ovaries and in the testis part of the ovotestes. The enriched biological process is shown as well as the *P*-value.

To investigate the effects of *Sall1* expression during early gonadal development, we induced its expression in the mouse ovary. For this purpose, we created a BAC construct to overexpress *Sall1* in somatic ovarian cells (Fig. 5A). The BAC contains the regulatory elements and the promoter of the *Wt1* gene, which is constitutively expressed in gonadal somatic cells (Zhao et al., 2014), but the gene is replaced by the coding sequence of *Sall1*. Through *PiggyBac* transgenesis, we integrated this construct into female mouse embryonic stem cells (mESC), which were subsequently used to generate transgenic mice through morula aggregation. In contrast to wild-type controls, mutant ovaries express *Sall1* in somatic cells, as indicated by the overlapping signal with *Foxl2*, a bona-fide marker for female somatic ovarian cells (Nicol et al., 2018) (Fig. 5B). However, at the phenotypic level, adult female mice did not show major morphological gonadal alterations and bred normally. Similarly, *Sall1*-overexpressing males develop normal testes and did not show any sign of reduced fertility (Fig. S15). This suggests that *Sall1*, by itself, is not sufficient to induce the development of testicular structures, nor to disrupt normal testis development.

To gain further insights into the molecular signatures of *Sall1* ovarian expression, we performed RNA-seq in gonads from mutants and littermate controls at E13.5. This analysis revealed around 400 deregulated genes where *Sall1* is the most significantly upregulated gene (Fig. 5C, Table S3). To understand the consequences of *Sall1* expression in female gonads, we compared the deregulated genes in the mutant ovaries with those specifically expressed in the testicular part of the ovotestis. We found 56 upregulated and 36 downregulated genes that are shared between the mutant mouse gonad and the testicular part of the mole ovotestis. Gene ontology analyses revealed no significant enrichment for the downregulated genes. However, the upregulated genes were enriched in terms related to the development of the kidney, a tissue in which *SALL1* is consistently expressed across mammalian species, as well as to ureteric bud morphogenesis and mesonephros development (Fig. 5D, Fig. S16).

The migration of somatic cells from mesonephros to gonad is a characteristic process of testis development, not occurring in ovaries (Martineau et al., 1997; Capel et al., 1999). However, gonads from female moles exhibited expression of migration markers, such as PDGFRa or MT1-MMP, suggesting that mesonephros-to-gonad migration might contribute to ovotestis formation (Carmona et al., 2009; Lupiáñez et al., 2012). However, no signs of migration were observed in the *Sall1*-overexpressing ovaries compared with those in wild type (Fig. S17), confirming that the expression of additional factors is required to induce cell migration from the mesonephros. Yet the expression of *SALL1* alone is sufficient to induce kidney-related gene programs, including mesonephros development, which are also observed in ovotestis development. Overall, our findings suggest that the expression of *SALL1* has been co-opted in mole ovotestes formation through the gain of specific enhancers, resulting in the recruitment of tissue-specific transcriptional programs.

## DISCUSSION

Across vertebrates, gonadal development is characterized by a remarkable evolutionary plasticity (Jiménez, 2009; Capel, 2017). This is highlighted by the development of ovotestes in moles, in which the development of a testicular region that increases the production of male hormones is fully compatible with a reproductive function (Jiménez et al., 1993). In previous studies, we demonstrated that mole ovotestis development is associated with a prolonged expression of *FGF9* through early gonadal development (Real et al., 2020). This heterochronic expression pattern delays the onset of female meiosis and creates a pro-testicular environment that is crucial for ovotestis development. Our transgenic experiment revealed that *SALL1* overexpression contributes to this transcriptional environment by activating gene expression programs. These programs are characterized by molecular signatures that are shared with other *SALL1*-expressing tissues, such as the kidney. Yet this ectopic program is not sufficient to trigger sex-reversal mechanisms, as indicated in phenotypical analyses. Therefore, it is plausible that *SALL1* may cooperate with other factors in ovotestis development and/or benefit from the pro-testicular environment that *FGF9* misexpression induces.

During evolution, genes are frequently co-opted for species-specific processes. These effects are often mediated by changes in the activity of regulatory elements that preserve the essential function of genes and, at the same time, allow a diversification of its expression in new tissues and cell types (Sanetra et al., 2005; McLennan, 2008; Holland, 2013; Jandzik et al., 2015; Chuong et al., 2016). Our analyses showed that mole *SALL1* enhancers were not able to recapitulate gonadal expression in mouse reporter assays. This could indicate that additional trans-acting factors are required for their activation, such as *IRF4* or *FOXP1.* Our analyses revealed that the mole enhancer sequences contain specific binding sites for these transcription factors, which also have higher levels of expression in ovotestes compared with mouse gonads. However, *SALL1* enhancers also display consistent activity in the mesonephros, a tissue that shares a common molecular origin with the gonad. Furthermore, the mesonephros is a known source of endothelial, myoid and supporting cells to the gonad (Burgoyne and Palmer, 1993; Brennan and Capel, 2004). Interestingly, the developing ovotestes of the mole, in contrast to female gonads of most mammalian species, show a prominent expression of migration markers (Carmona et al., 2009; Lupiáñez et al., 2012). Thus, the formation of mole ovotestis may involve the recruitment of cells from the adjacent mesonephros, which may explain the activity of mole enhancers in this tissue. Interestingly, the mesonephric activation of *SALL1* is driven by several enhancers, thus resembling the functional redundancy of CREs that has been described at multiple developmental loci (Osterwalder et al., 2018). Such cooperative activity has been proposed to arise by an initial gain in transcription factor binding sites that is progressively stabilized through the recruitment of additional sites at other elements, giving the capacity to these elements to evolve redundant functions (Spitz and Furlong, 2012). We observed a similar mechanism in the regulatory landscape of *SALL1*, where several of the enhancers share binding sites for transcription factors specifically expressed in the testicular part of the ovotestes. Furthermore, this pattern of transcription factor binding is highly distinct from other mammal species, such as mouse or shrew, suggesting the capacity of these non-coding elements to evolve.

TAD structures serve as a spatial scaffold in which regulatory elements interact with their cognate genes, thus representing the existence of large 3D regulatory landscapes contributing to the specificity of gene expression. These domains have been suggested to represent a fertile ground for the evolution of gene expression (Hoencamp et al., 2021; Anania and Lupiáñez, 2020; Rowley and Corces, 2018). Previous studies have demonstrated that TADs impose important constraints during evolution, as genomic rearrangements are more prone to occur at boundaries, preserving TADs as entire regulatory units (Krefting et al., 2018). However, genomic rearrangements that reorganize TADs can be also associated with changes in gene expression that might induce the evolution of traits (Ghavi-Helm et al., 2019). This has been recently exemplified with the ectopic activation of the PCP pathway being linked to the development of enlarged fins in skates, and also in moles, where genomic rearrangements affecting the *FGF9* and *CYP17A1* TADs are associated with intersexuality (Real et al., 2020; Marlétaz et al., 2023). In contrast, our current study also highlights that the evolution of CREs within conserved TADs is another relevant mechanism for evolution. This is indicated by the striking conservation of TAD organization at the *SALL1* TAD, which is characterized by a remarkable internal evolution of CREs. These results are consistent with previous observations and further reinforce the idea that TADs might serve as a scaffold for the evolution of gene pleiotropy (Franke and Gómez-Skarmeta, 2018; Acemel et al., 2017). In summary, our results suggest the co-option of *SALL1* in mole ovotestis development, through regulatory changes that occur despite a striking conservation of TAD organization. This highlights the multilayered nature of gene regulation and how changes at different levels may serve as a driving force for the evolution of traits.

## MATERIALS AND METHODS

### Animal models

Adult, infant or embryonic specimens of the Iberian mole *Talpa occidentalis* were used with annual permission from the Andalusian Environmental Council granted to Prof. Rafael Jiménez. The animals were captured alive in poplar groves plantations in Santa Fe, Chauchina and Fuentevaqueros (Granada province, southern Spain) using an efficient trapping system as described in a previous publication (Barrionuevo et al., 2004) and handled according to the guidelines and approval of the Ethical Committee for Animal Experimentation of the University of Granada.

Hedgehogs (*Atelerix albiventris*) were maintained in the LANE animal facility at the University of Geneva and were sampled under the experimentation permit GE24/33145 approved by the Geneva cantonal veterinary authorities, Switzerland.

Shrews (*Sorex araneus*) were trapped in wooden traps and euthanized with an isoflurane overdose followed by open-heart perfusion (see Lázaro et al., 2018 for details) in Möggingen, Germany, under permit number 35-9185.81/G-11/21 to D.K.N.D.

*lacZ* transgenic mice were created at the Lawrence Berkeley National Laboratory (LBNL, CA, USA), which is reviewed and approved by the LBNL Animal Welfare Committee. Transgenic mice were housed at the Animal Care Facility (the ACF) at LBNL. All transgenic experiments were performed in accordance with national laws and approved by the national and local regulatory authorities. Mice were monitored daily for food and water intake, and animals were inspected weekly by the Chair of the Animal Welfare and Research Committee and the head of the animal facility in consultation with the veterinary staff. The LBNL ACF is accredited by the American Association for the Accreditation of Laboratory Animal Care International (AAALAC).

The experiments for *Sall1* overexpression transgenic mice were performed as approved by LAGeSo Berlin under license numbers G0346/13 and G0247/13. Transgenic experiments were performed using mouse embryonic stem cells (mESCs) from a C57BL/6J or C57BL/6J-129 hybrid background. For RNA-seq and ChIP-seq experiments, gonads from wild-type CD1 mice were used.

### Histological and immunostaining analyses

Gonads from adult animals, infants and embryos were fixed in 4% PFA and embedded in paraffin wax. The embedded samples were sectioned at 5μm and stained with Hematoxylin and Eosin according to standard protocols.

For protein spatio-temporal detection experiments, indirect immunofluorescence was used. In brief, sample slides were incubated overnight with the primary antibody at a dilution according to the manufacturer's instructions. Next, samples were incubated with specific Alexa secondary antibodies 488 and 568 together with DAPI for 1 h at room temperature. Slides were then mounted in fluoromount-G solution (SouthernBiotech) and pictures were taken either with a laser confocal Zeiss LSM700 or a Zeiss Axiovert 200 M microscope. Primary antibodies and working dilutions were as follows: mouse anti-SALL1 (Abcam ab41974, dilution 1:100), goat anti-FOXL2 (Abcam ab5096, dilution 1:200) and rabbit anti-SOX9 (Cell Signaling 82630, dilution 1:200).

### RNA isolation and cDNA synthesis

Total RNA was extracted from adult ovaries and kidney from hedgehog (Atelerix albiventris) and shrew (Sorex araneus) using RNeasy Mini Kit (Quiagen, 74106) according to the manufacturer's instructions. In short, the tissues were homogenized in RTL buffer supplemented with β-Mercaptoethanol and applied to spin columns. Genomic DNA was removed using RNase-Free DNase Set (Quiagen, 79254). Eluted RNA quality and concentration were measured using NanoDrop 2000 UV spectrophotometer.

RNA (1 µg) per sample was used for reverse transcription into cDNA using SuperScript IV First-Strand Synthesis System (Invitrogen, 18091050) according to the manufacturer's instructions. In short, random hexamer primers were annealed to template RNA and RNA was reverse transcribed into cDNA. Finally, RNA was removed using RNAse H and a reverse transcription reaction was used for RT-qPCR.

### RT-qPCR

*SALL1* and *FOXL2* mRNA levels were quantified by RT-qPCR for two biological replicates each in technical triplicate. RT-qPCRs were performed using 2× Blue S'Green qPCR Kit Separate Rox (Biozym, 331416) according to the manufacturer's instructions with 27.5 ng cDNA and 100 nM of each primer. All experiments were performed on QuantStudio 7 Flex system (Thermo Fisher).

Expression levels were normalized to *RPS9* mRNA. The 2-ΔΔCt method was used for analysis of relative *SALL1* and *FOXL2* expression levels. A one-tailed *t*-test was applied in these experiments.

### ChIP sequencing

Gonads from E13.5 mouse embryos were fixed using 1% formaldehyde and subsequently snap-frozen and stored at −80°C. Chromatin immunoprecipitations were performed using the iDeal ChIP-seq Kit for Histones (Diagenode, C01010051) according to the manufacturer's instructions. Briefly, whole fixed gonads were lysed and subsequently sonicated using a Bioruptor (45 cycles, 30 s on, 30 s off, at high power) in the provided buffers. Sheared chromatin (5 µg per immunoprecipitation) was then used with 1 µg of the following specific histone antibodies: anti-H3K4me3 (Millipore, 07-473), anti-H3K4me1 (Diagenode, C15410037) and anti-H3K27ac (Diagenode, C15410174). The samples were sequenced using Illumina HiSeq technology according to standard procedures. Mapping was performed with the STAR v2.6.1d software41 using settings to enforce unspliced read mapping (--alignEndsType EndToEnd --alignIntronMax 1 --outFilterMatchNminOverLread 0.94). Finally, de-duplication was performed via bamUtil (version 1.0.14; option –rmDups, https://github.com/statgen/bamUtil/releases). Previous published ChIPseq data from mole developing gonads (Real et al., 2020) were used to call putative enhancer regions.

### Enhancer calling and conservation

Calling of putative enhancer regions was performed for mole and mouse via the software CRUP with replicates merged beforehand. CRUP software combines profiles from three histone marks, H3K4me3, H3K4me1 and H3K27ac, to define active enhancers. Enhancer regions with a distance ≤200 bp were merged. To reduce outlier effects in enhancer probability scores, a smoothing over five bins of 100 bp was applied. In line with the original CRUP results, the probability of an enhancer region is defined as the, now smoothened, maximum score of the 100 bp bins overlapping the enhancer. For the analysis of enhancer conservation, mole enhancer regions were lifted-over to the mouse genome (mm9). By definition, only those regions overlapping a conserved sequence block can be lifted and therefore depend on genome alignment settings. Here, we performed a sensitive pair-wise one-to-one genome alignment using LAST with automated training of optimal alignment parameters. In cases where an enhancer overlaps a conserved block partially, the respective non-conserved boundary is interpolated by the distance to the closest conserved block. Accordingly, the size of the lifted enhancer region in mm9 will be approximately the same as the one of the respective mole enhancer. Nevertheless, to exclude artefacts, lifting is only accepted if the ratio of mole enhancer length/lifted length<1.5. We define an enhancer sequence as conserved if the enhancer could be lifted successfully. In addition, we define an enhancer as conserved in enhancer function if the mole enhancer overlaps a mouse enhancer irrespective of tissue-specificity.

### Transcriptomic analyses

For gene expression analysis, gonads from adult mice and embryos at E13.5 were dissected, and RNA was extracted from these samples using the RNeasy Mini Kit (QIAGEN) according to the manufacturer's instructions. For mole gonads, previously published RNA-seq data were used (Real et al., 2020). The samples were sequenced using Illumina HiSeq technology according to standard procedures. Read mapping was performed with the STAR v2.6.1d software (Dobin et al., 2013). Read counts were created using the R function ‘summarizeOverlaps’ and normalized to RPKM based on the number of uniquely mapped reads. For the analysis of differential expression between samples, the DESeq2 tool was used with default settings (Anders and Huber, 2010).

### Definition of female testis part specific regions

In order to prioritize enhancers by their potential relevance to the testis part of the tissue, we first ranked enhancer regions by the difference in enhancer probability (score in the testis part versus mean of scores in the testis+ovary part). We defined the putative target gene of each enhancer as that with the closest transcriptional start site to the center of the enhancer region within the same TAD. Based on the differential expression analysis (testis part versus testis+ovary part), each target gene is ranked by specific expression in ovotestis (log2 fold-change). Finally, enhancers are ranked jointly for functional importance in the testis part of the ovotestis by the mean rank of probability score and the rank of the putative target gene.

### Transcription factor binding motif enrichment analysis

The five *SALL1* enhancer sequences from Talpa occidentalis were lifted over to the genomes of mouse (UCSC:mm39) and *Sorex araneus* (UCSC:SorAra2.0) based on pair-wise genome comparisons. In case of partial conservation, enhancer boundaries were approximated given the sizes of the remaining non-conserved parts in Talpa.

For each enhancer sequence, transcription factor (TF) binding affinities were computed via TRAP7 for all TransFac motifs (release TFP_2022.2). In the case of TFs represented by multiple motifs, only the one with the smallest *P*-value was kept. In addition, TFs not expressed in gonads (RPKM<3) were discarded. Finally, for each species, TFs were ranked by the mean -log(*P*-value) across the group of five enhancers. To avoid artefacts introduced by non-significant binding affinities, *P*<0.05 was set as a cutoff. As a consequence, the computed mean should roughly correlate with the number of TF binding sites. Typically, only a subset of enhancers shares significant binding affinity for a specific TF (Table S2, column 6) that shows a sufficient expression (Table S2, column 4) in at least one gonadal tissue. Original affinity *P*-values for enhancers 1-5 are listed in the last column of Table S2.

### Hi-C

Previously published datasets from mole embryonic limb buds and adult ovotestes were used to inspect the SALL1 regulatory domain (Thomas-Chollier et al., 2011). Maps were visualized with Juice box software (Durand et al., 2016a).

Mouse Hi-C was obtained from publicly available high-resolution datasets from neuronal progenitor cells (NPCs) (Bonev and Cavalli, 2016). Maps were visualized with Juice box software (Durand et al., 2016b).

### 4C sequencing

Embryonic tissues were dissociated with trypsin, filtered through a cell strainer to obtain a single cell suspension and subsequently fixed in 2% formaldehyde. Mouse embryonic stem cells (mESCs) were detached from culture plates and fixed in the same way. Cells were counted and five million cells were snap-frozen and stored at −80°C until processing.

4C-seq libraries were prepared according to standard protocols (van de Werken et al., 2012). For the initial digestion, NlaIII was used in *SALL1* experiments and BfaI was used in ITR-BAC ES cells. For the second digestion, DpnII was used for all experiments. A total of 1.6 mg of each library was amplified by PCR for each viewpoint with primers listed in Table S4. The libraries were sequenced using Illumina HiSeq technology according to standard procedures. Raw reads were pre-processed and mapped to the reference genome (talOcc4) using BWA (Li and Durbin, 2010). Finally, reads were summarized and normalized by coverage (RPM) for each fragment generated by neighboring restriction enzyme sites. The viewpoint and its flanking fragments (1.5 kb upstream and downstream) were removed for data visualization and a window of 10 fragments was used to smoothen the data.

The mouse virtual 4C profile was derived from a genome-wide Hi-C map from NPCs (Thomas et al., 2003) by first extracting the intrachromosomal contact maps for the chromosomes of interest using Juicer tools v0.7.5 (Durand et al., 2016b) (KR normalized, MAPQ>=30, 5 kb resolution). Afterwards, only map entries with at least one bin overlapping the viewpoint [chr8:89,044,162 (*Sall1*) on mm10] were used for the virtual 4C profile.

### *lacZ* reporter assay in transgenic mice

*lacZ* transgenic mouse reporter assays were conducted as described previously (Osterwalder et al., 2022). Briefly, enhancer sequences were amplified by PCR from mole genomic DNA using primers listed in Table S4. PCR products were cloned into a vector containing a minimum promoter, hsp68, in front of the *lacZ* gene. For microinjection into fertilized eggs, plasmid DNA was linearized with PacI and purified using Montage PCR filter units and Micropure EZ column (Millipore). For pronuclear injection of FVB embryos, DNA was diluted to a final concentration of 1.5-2 ng/µl and used in accordance with standard protocols approved by the Lawrence Berkeley National Laboratory. Embryos were harvested at embryonic day 13.5, dissected and fixed in 4% paraformaldehyde (PFA). Tissues were stained for 24 h with freshly prepared staining solution, washed and post-fixed in 4% PFA.

### BAC transgenesis for overexpression of *Sall1*

*SALL1*-coding sequence (CDS) was amplified from a vector containing the cDNA mouse sequence (Origen, MC203471) with specific primers compatible with the attB gateway recombination system (Invitrogen). Through the gateway system, the generated product was introduced into a modified Wt1-BAC, containing piggyBac DNA transposon elements, as well as attL docking sites. The Wt1-BAC vector was kindly provided by Dr Koopman and its further modification was performed according to their previously published method (Zhao et al., 2014). After introduction of the *SALL1* minigene, a eukaryotic antibiotic resistance (dual Neomycin-Kanamycin cassette) was introduced into the BAC vector through recombineering for transfection into ES cells according to the protocol previously described (Wang et al., 2006). Primers are listed in Table S4.

### BAC transfection into female ES cells

Blastocysts from C57BL/6J mice were used to derive mouse embryonic stem cells (mESCs) by growing them with culture medium supplemented with leukemia inhibitory factor (LIF), as well as FGF/Erk and Gsk3 pathway inhibitors (2i). The derived mESCs were genotyped for sex and a female line was expanded through co-culture with mouse embryonic fibroblasts (MEFs) for further experiments.

Female mESCs were co-transfected with 3 µg piggybac transposase and 500 ng of the modified Wt1-*SALL1*-piggyBac-Neo-BAC using Lipofectamine LTX (Invitrogen), as described in a previous publication (Rostovskaya et al., 2012). After Geneticin-G418 selection (250 µg/ml) for 5 to 10 days, clones were picked and checked for successful BAC integration with three genotyping PCRs. A primer pair targeting each piggybac ITR (5′ITR and 3′ITR) was used as positive control, while a primer pair targeting the BAC vector was used as negative control to confirm integration mediated by transposition, instead of random insertion. Positive clones were expanded and additional genotyping was carried out by 4C-seq, to confirm genomic integrations site, as well as number of integrations, as described previously (van de Werken et al., 2012).

### Gene ontology analyses

For Gene Ontology (GO) terms, enrichment analysis PANTHER software was used (Thomas et al., 2003), selecting all the common upregulated genes for the testis part of the ovotestes and in the *Sall1*-overexpressing mouse mutants. A total of 56 genes was evaluated. No significant enrichment was found for the downregulated genes.

## Supplementary Material

10.1242/develop.201562_sup1 Supplementary information
