# Supplementary material for: Induction of kidney-related gene programs through co-option of SALL1 in mole ovotestes
Source: Development. 2023 Sep 5;150(17):dev201562. doi: 10.1242/dev.201562 (PMC10499028; doi:10.1242/dev.201562)
Supplement: Supplementary information [file develop-150-201562-s1.pdf]

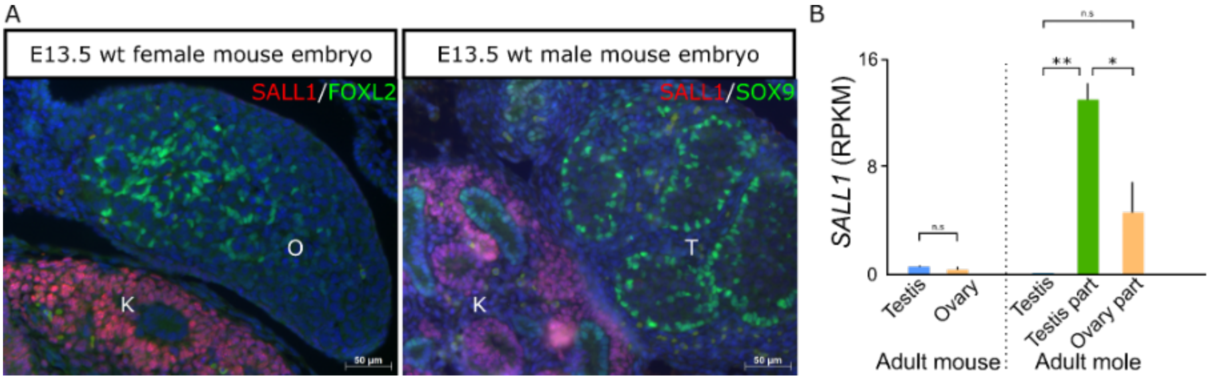

Fig. S1. *Sall1* expression pattern in mouse gonads.

A. Immunostainings of SALL1 (red) and FOXL2 and SOX9 (green) as markers of somatic female and male cells, respectively. O: ovary, T: testis, K: kidney. Note the absence of SALL1 positive cells in the embryonic gonads but the specific expression in the adjacent kidney. Scale bars: 50 µm.

B. RPKMs quantification from RNA-seq data of adult gonads in mouse and mole. Expression levels in mouse are lower compared to mole and not sex specific.

Development • Supplementary information

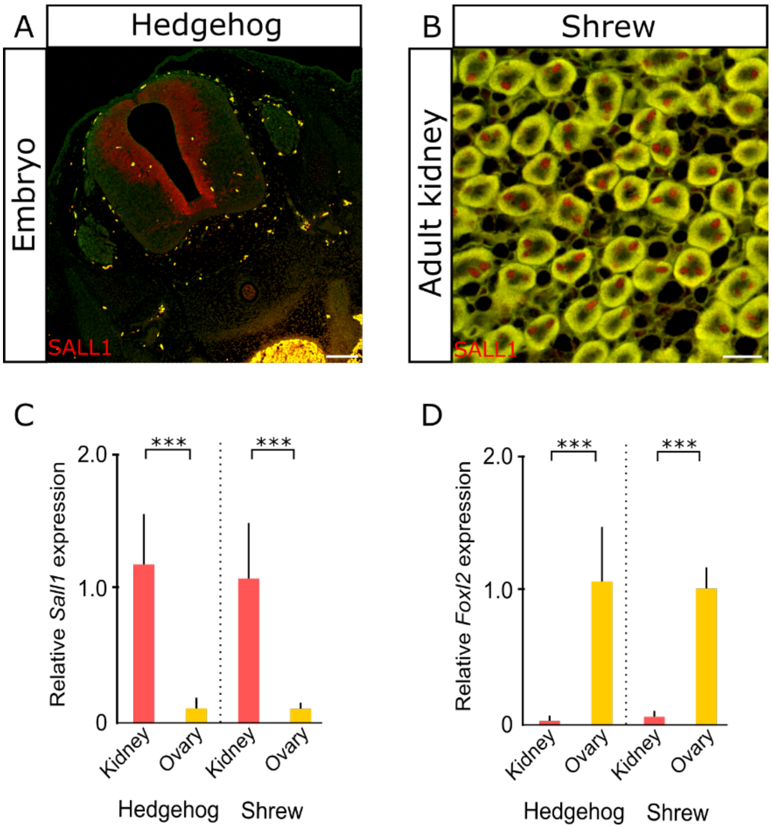

Fig. S2. *SALL1* expression in *Eulipotyphla* species.

A. Immunostaining of SALL1 in transversal sections of an early hedgehog embryo from the *Atelerix albiventris* species. SALL1 is highly expressed in the neural tube, a well-known tissue for SALL1 expression. Scale bar: 100 µm.

B. Immunostaining of SALL1 in adult kidneys from the common shrew, *Sorex araneus*. Note the specificity of the antibody to the nucleus of the renal tubular cells. Scale bar: 20 µm.

C. RT-qPCRs for *SALL1* expression in adult ovaries and kidneys from hedgehogs (*Atelerix albiventris*) and shrews (*Sorex araneus*). Shown is relative *SALL1* expression normalized to *RPS9*. Data is presented as mean ± SD and p-values are indicated as *$p \leq 0.05$, **$p \leq 0.01$, ***$p \leq 0.001$.

D. RT-qPCRs for *FOXL2* expression in adult ovaries and kidneys from hedgehogs (*Atelerix albiventris*) and shrews (*Sorex araneus*). Shown is relative *FOXL2* expression normalized to *RPS9*. Data is presented as mean ± SD and p-values are indicated as *$p \leq 0.05$, **$p \leq 0.01$, ***$p \leq 0.001$.

Development • Supplementary information

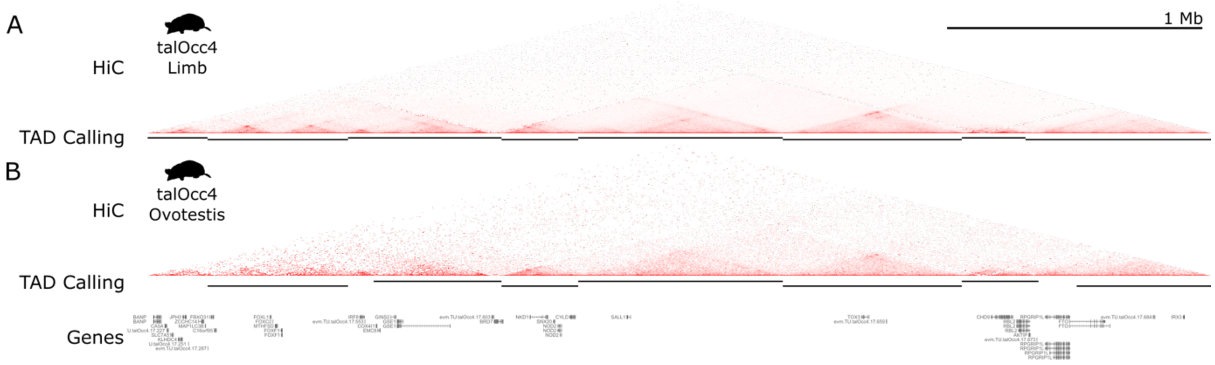

Fig. S3. Hi-C map comparison between limb and ovotestis

A. Hi-C maps at high resolution from embryonic limbs with the corresponding TAD calling (black bars) underneath.

B. Hi-C maps from adult ovotestis with the corresponding TAD calling (black bars) underneath. Note the conservation of the *SALL1* TAD domain between tissues.

Development • Supplementary information

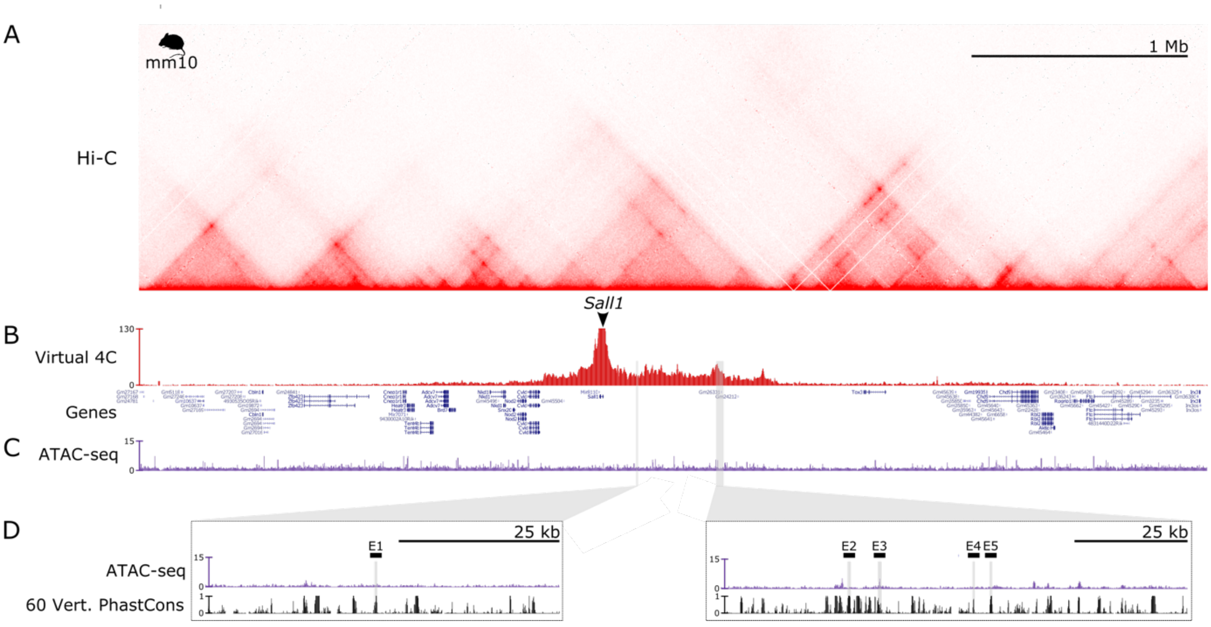

Fig. S4. Regulatory domain of *Sall1* in mouse.

A. Hi-C map from Neural Progenitor Cells (NPCs) denotes the domain of *Sall1* in a large gene desert.

B. Virtual 4C-seq analysis from NPCs Hi-C maps with *SALL1* promoter as viewpoint. Note high interaction frequency between the gene promoter and the surrounding 1Mb desert clearly demarcating the *Sall1* regulatory domain. The domain is strikingly conserved between cell types and species.

C. ATAC-seq track from mouse embryonic kidneys at E14.5 to identify regulatory regions in this tissue.

D. Zoom-in on the two equivalent regions where the mole enhancers were identified. Homologous regions are marked as gray bars and labeled as E1-5. Consistent with our enhancer activity results, enhancer 3 (E3) coincides with an ATAC-seq peak in kidneys.

Development • Supplementary information

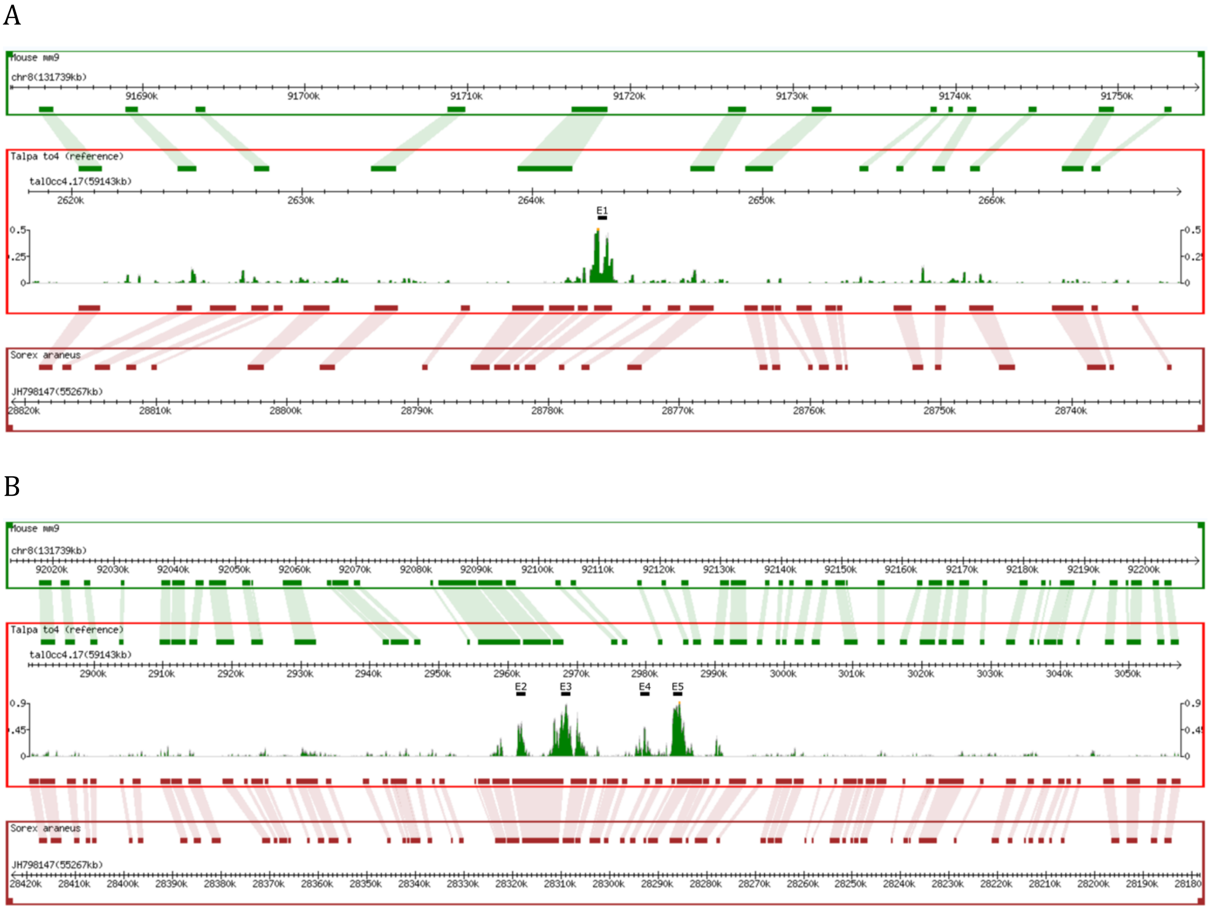

Fig. S5. Synteny of the enhancer regions.

A. Alignment of syntenic blocks for the enhancer region 1 (E1) against the mouse genome (upper panel) and against the shrew genome (*Sorex araneus*, lower panel). Visualization with Gbrowse[46].

B. Alignment of syntenic blocks for the cluster of enhancers (E2-E5) against the mouse genome (upper panel) and against the shrew genome (*Sorex araneus*, lower panel). Visualization with Gbrowse[46].

Development • Supplementary information

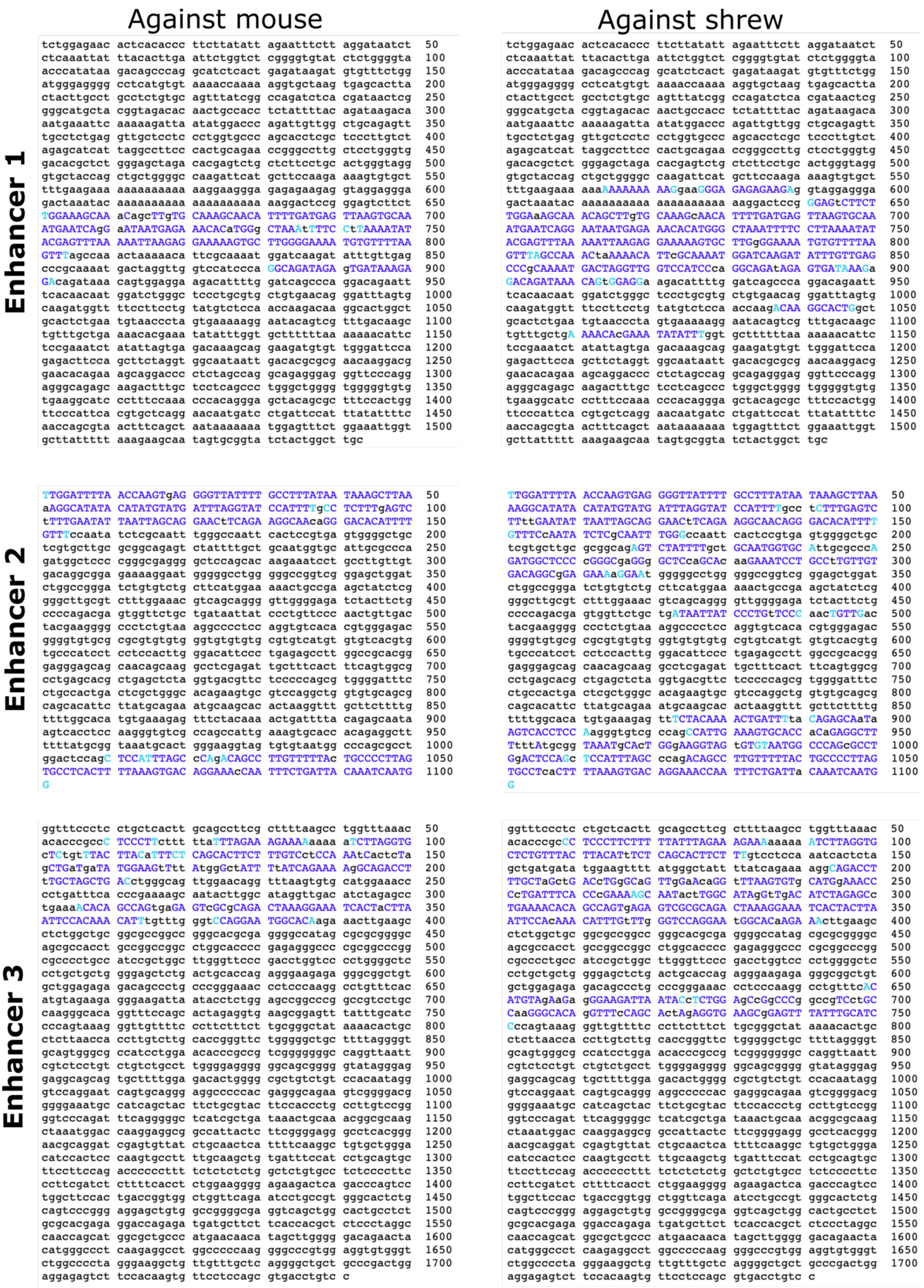

Fig. S6. Sequence alignments for the individual enhancers 1 to 3.

The conserved nucleotides are highlighted in blue and capitalized. Light blues nucleotides denote the beginning and end of the homology sequence.

Development • Supplementary information

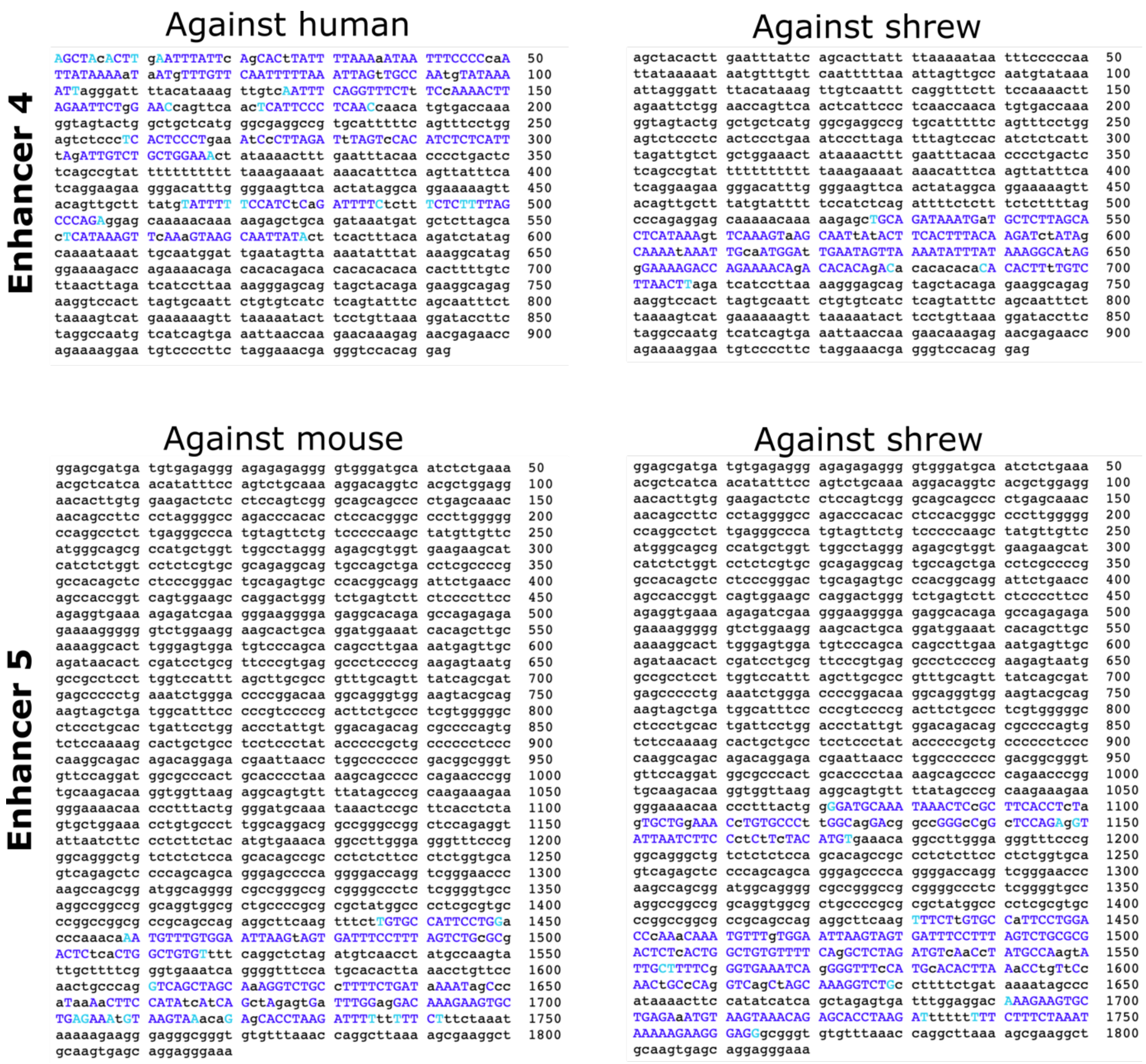

Fig. S7. Sequence alignments of the individual enhancers 4 and 5.

The conserved nucleotides are highlighted in blue and capitalized. Light blues nucleotides denote the beginning and end of the homology sequence. Note that for enhancer 4 there was no homology in the sequence compared to mouse, human was used instead.

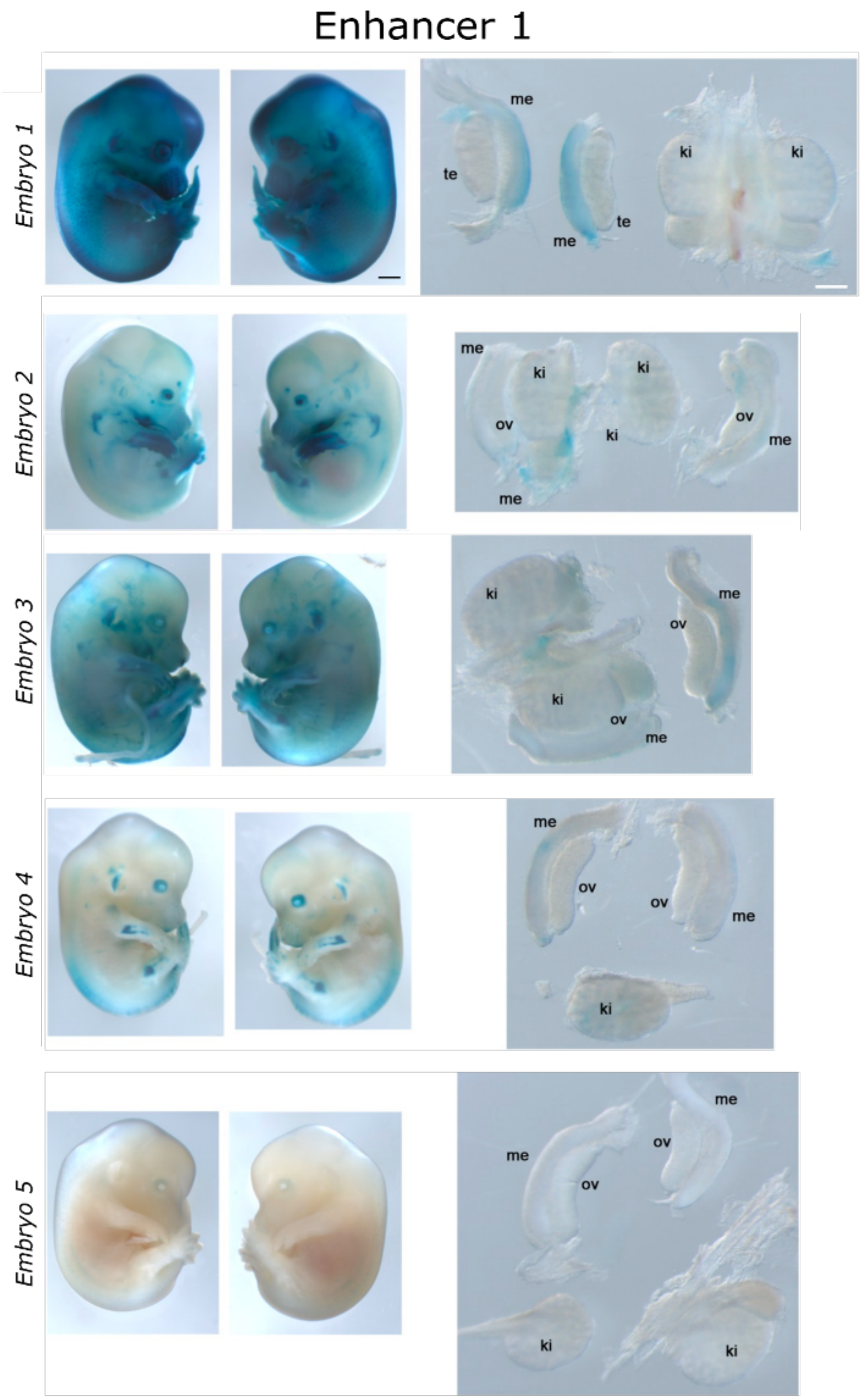

Fig. S8. LacZ enhancer reporter assay for Enhancer 1.

All embryos analyzed for this enhancer are depicted. Entire embryos at E13.5 as well as the dissected urogenital tracts are displayed. me: mesonephros, te: testes, ov: ovaries, ki: kidneys. Four out of five embryos showed mesonephros-specific staining. Black scale bars: 1000 µm, white scale bars: 100 µm.

Development • Supplementary information

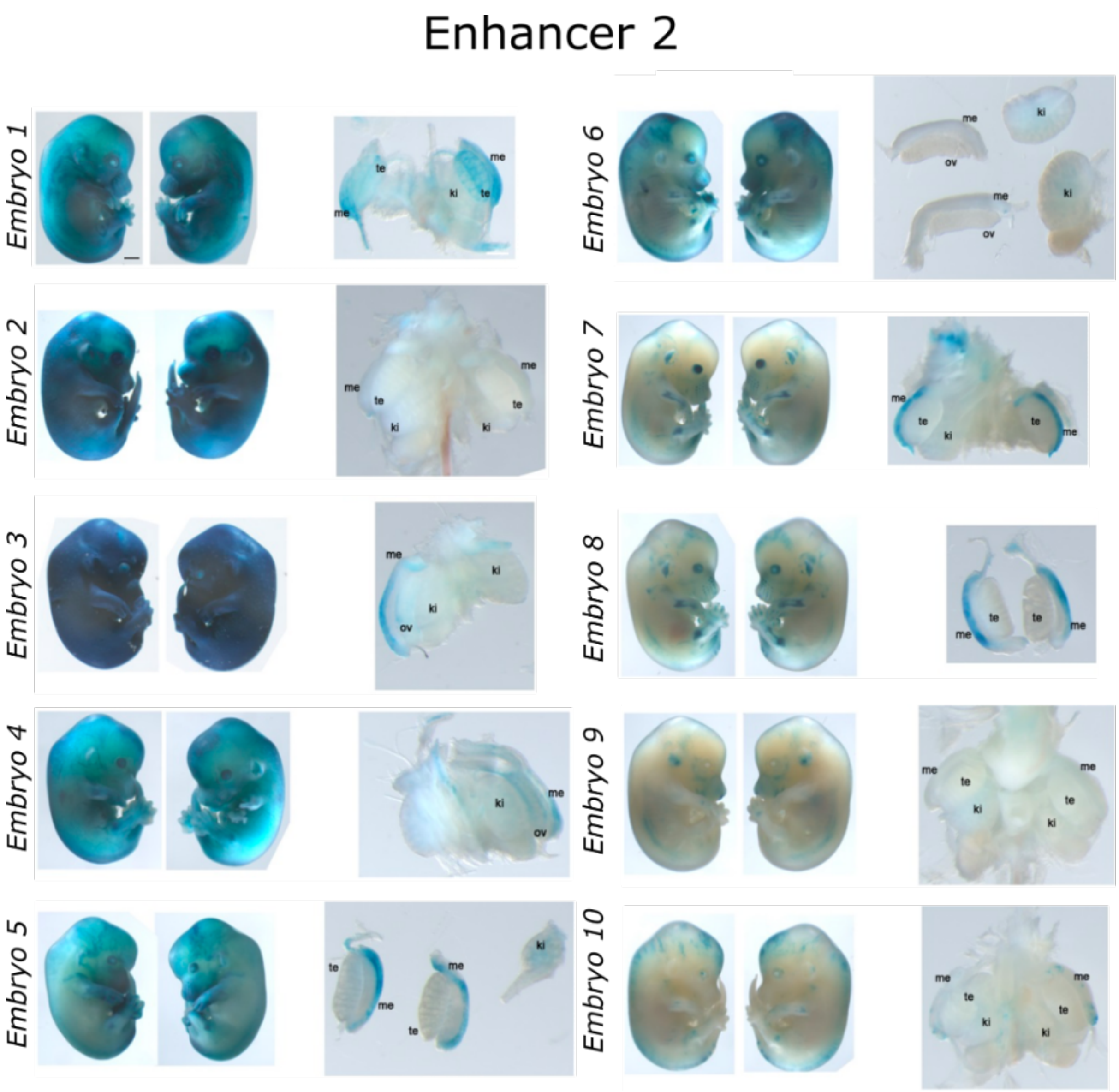

Fig. S9. LacZ enhancer reporter assay for Enhancer 2.

All embryos analyzed for this enhancer are depicted. Entire embryos at E13.5 as well as the dissected urogenital tracts are displayed. Me: mesonephros, te: testes, ov: ovaries, ki: kidneys. Seven out of ten embryos showed mesonephros-specific staining. Black scale bars: 1000 µm, white scale bars: 100 µm.

Development • Supplementary information

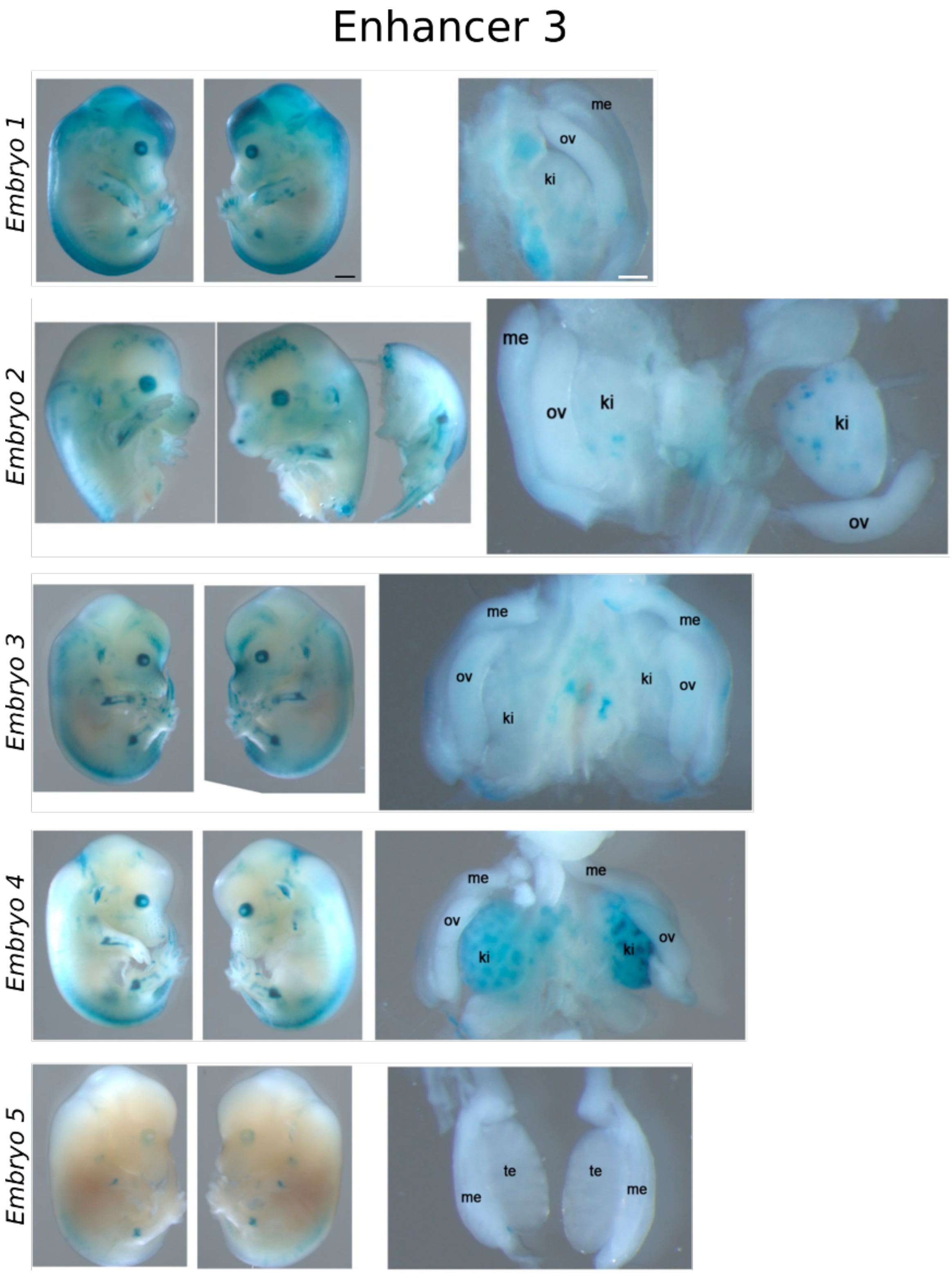

Fig. S10. LacZ enhancer reporter assay for Enhancer 3.

All embryos analyzed for this enhancer are depicted. Entire embryos at E13.5 as well as dissected urogenital tracts are displayed. Me: mesonephros, te: testes, ov: ovaries, ki: kidneys. Three out of five embryos showed kidney-specific staining. Black scale bars: 1000 µm, white scale bars: 100 µm.

Development • Supplementary information

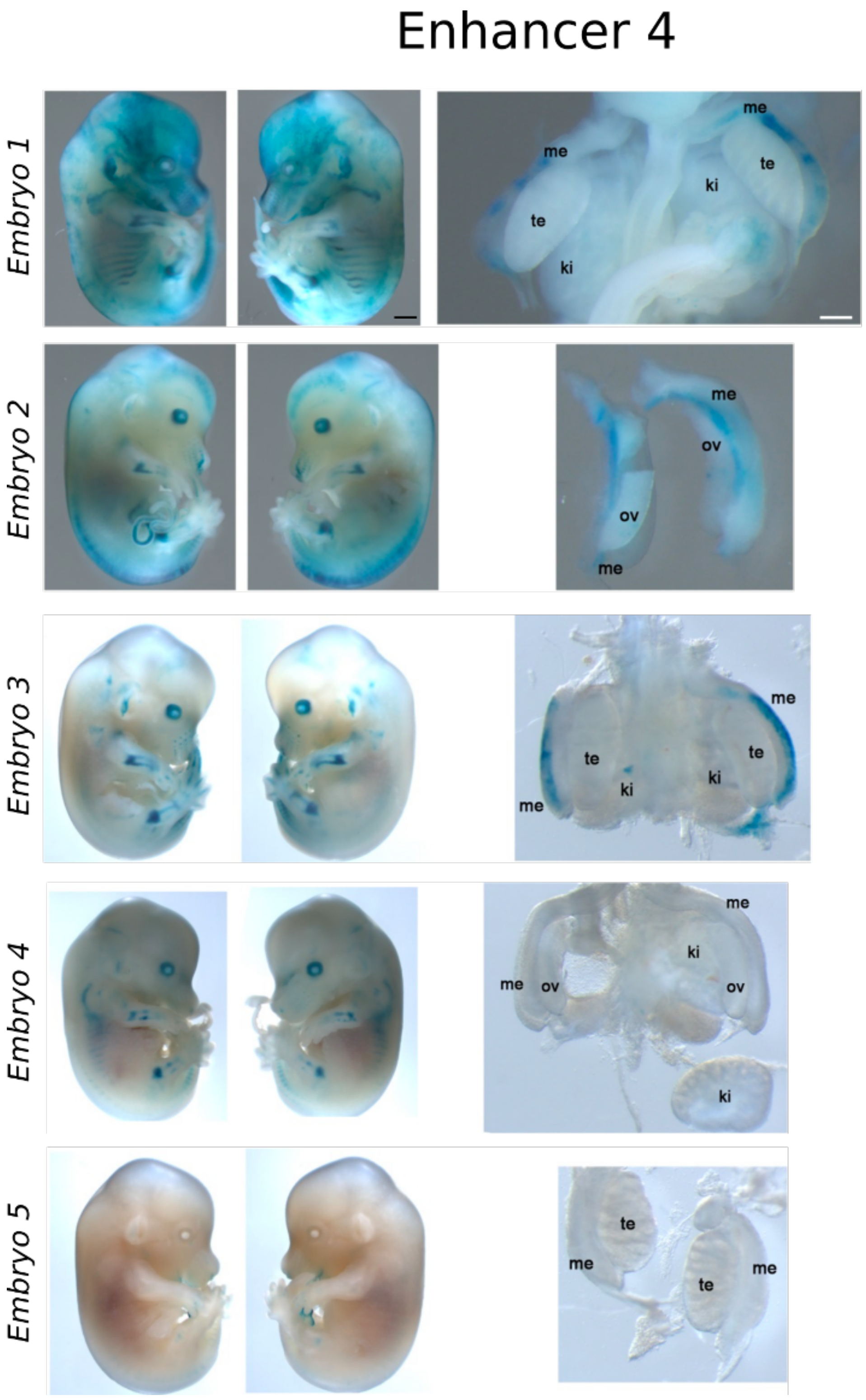

Fig. S11. LacZ enhancer reporter assay for Enhancer 4.

All embryos analyzed for this enhancer are depicted. Entire embryos at E13.5 as well as the dissected urogenital tracts are displayed. Me: mesonephros, te: testes, ov: ovaries, ki: kidneys. Three out of five embryos showed mesonephros-specific staining. Black scale bars: 1000 µm, white scale bars: 100 µm.

Development • Supplementary information

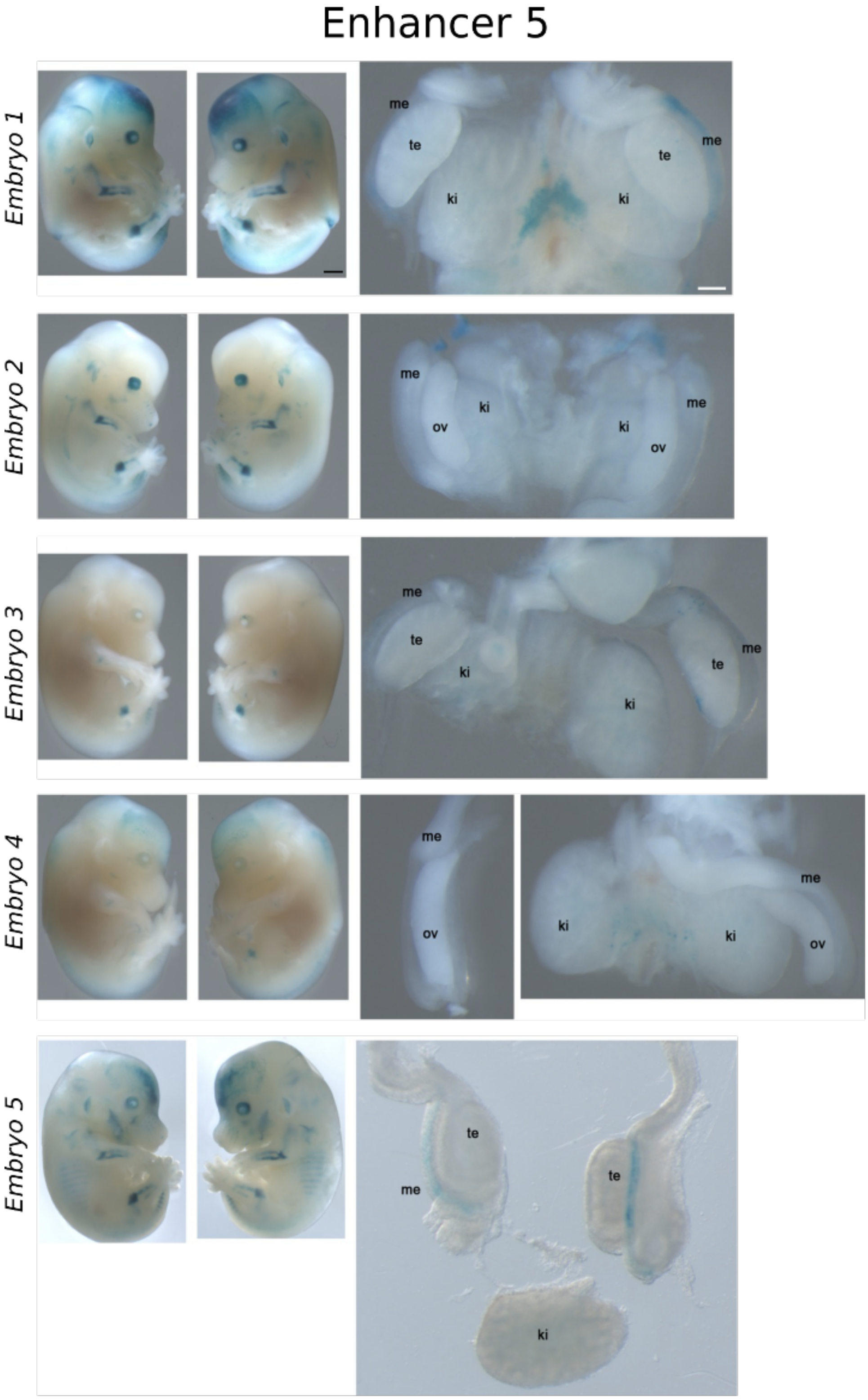

Fig. S12. LacZ enhancer reporter assay for Enhancer 5.

All embryos analyzed for this enhancer are depicted. Entire embryos at E13.5 as well as the dissected urogenital tracts are displayed. Me: mesonephros, te: testes, ov: ovaries, ki: kidneys. Two out of five embryos showed mesonephros-specific staining. Black scale bars: 1000 µm, white scale bars: 100 µm.

Development • Supplementary information

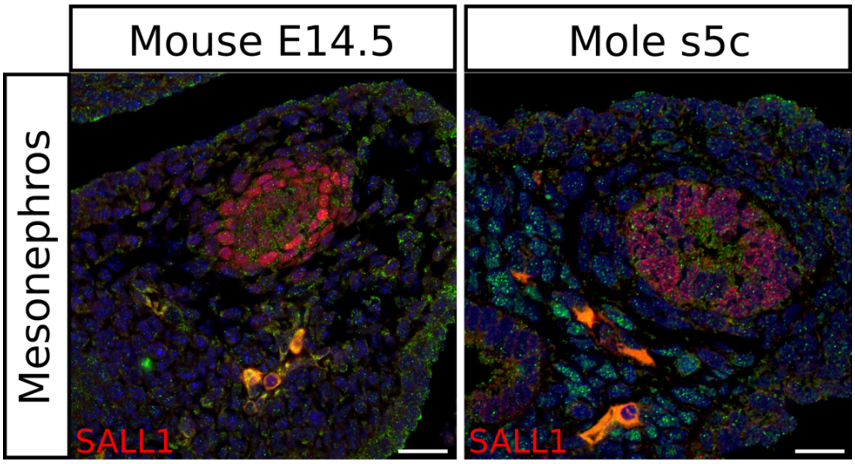

Fig. S13. SALL1 expression in mesonephros

SALL1 is detected in the mesonephros duct of mouse at E14.5 and at equivalent stages in moles (s5c). Scale bars: 20 µm.

Development • Supplementary information

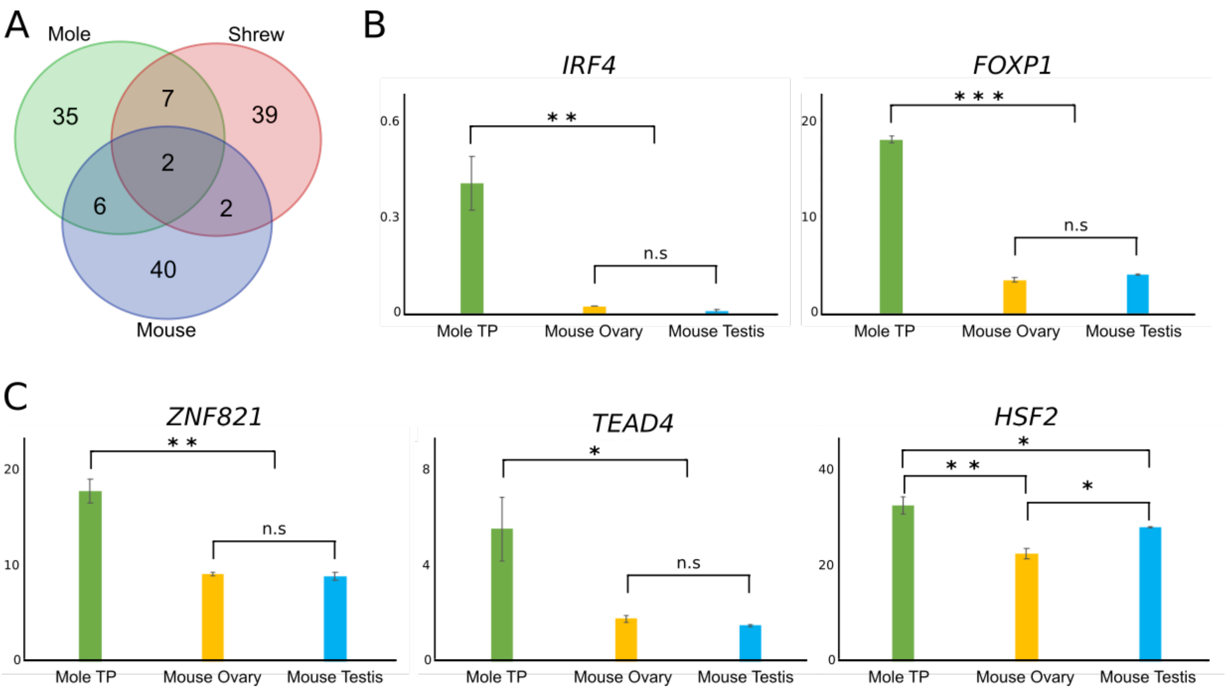

Fig. S14. Comparative analyses of transcription factor binding motifs and expression.

A. Venn diagram showing the number of shared transcription factor binding motifs among the top 50 motifs found in mole, shrew and mouse sequences (**Supplementary Table 2**). Note the limited conservation, emphasizing the sequence divergence observed among species.

B, C. Expression levels in RPKM of transcription factors with top-ranked motif bindings sites in the mole enhancer sequences. The mole TP (testicular part) of the female ovotestis at P7 is compared with the mouse ovary and testis at E13.5. Note the upregulation of these 5 transcription factors when compared to mouse gonads. Data is presented as mean ± SD and p-values are indicated as $*p \leq 0.05$, $**p \leq 0.01$, $***p \leq 0.001$.

Development • Supplementary information

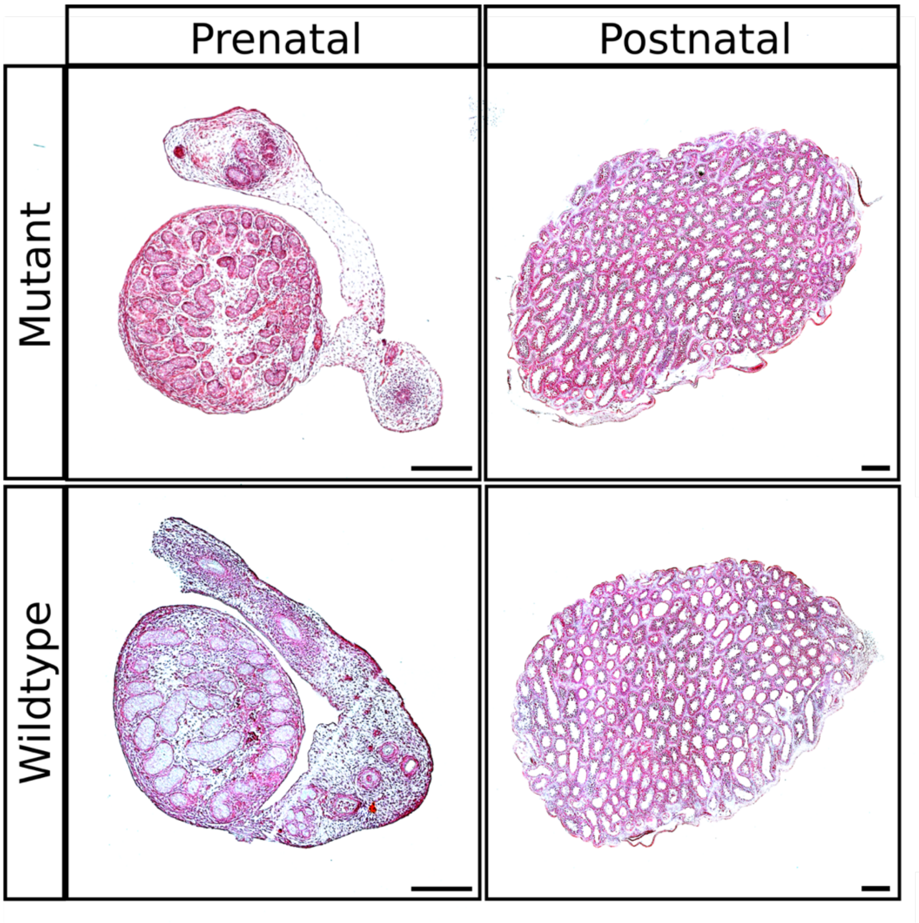

Fig. S15. Morphology of *Sall1*-overexpressing testes during gonad development.

Hematoxylin-eosin stanning of mutant overexpressing-*Sall1* and wildtype controls testes before and after birth. There are no differences in size, tissue structure or cell composition between mutants and controls. Scale bars: 200 µm.

Development • Supplementary information

A

GO biological process complete	Mus musculus (REF) #	upload_1 (▽ Hierarchy NEW! ⓘ) #	expected	Fold Enrichment	+/-	raw P value	FDR
metanephric nephron morphogenesis	18	3	.04	67.89	+	1.79E-05	4.03E-02
↳metanephros morphogenesis	24	3	.06	50.92	+	3.89E-05	4.09E-02
↳animal organ development	3306	20	8.12	2.46	+	6.24E-05	4.68E-02
↳system development	3813	25	9.36	2.67	+	8.56E-07	1.35E-02
↳multicellular organism development	4559	26	11.19	2.32	+	1.01E-05	2.64E-02
↳urogenital system development	368	7	.90	7.75	+	3.46E-05	4.20E-02
↳animal organ morphogenesis	1057	11	2.59	4.24	+	4.59E-05	4.02E-02
↳anatomical structure morphogenesis	2337	19	5.74	3.31	+	1.50E-06	1.18E-02
↳nephron development	136	5	.33	14.98	+	2.45E-05	3.51E-02
positive regulation of branching involved in ureteric bud morphogenesis	23	3	.06	53.13	+	3.47E-05	3.90E-02
↳regulation of branching involved in ureteric bud morphogenesis	25	3	.06	48.88	+	4.35E-05	4.04E-02
↳regulation of morphogenesis of a branching structure	66	4	.16	24.69	+	2.61E-05	3.43E-02
↳regulation of multicellular organismal process	2989	19	7.34	2.59	+	5.28E-05	4.38E-02
ventricular septum development	82	4	.20	19.87	+	5.88E-05	4.63E-02
↳cardiac ventricle development	145	6	.36	16.86	+	1.84E-06	9.66E-03
↳cardiac chamber development	192	6	.47	12.73	+	8.77E-06	2.76E-02
developmental growth involved in morphogenesis	153	5	.38	13.31	+	4.22E-05	4.16E-02
anatomical structure formation involved in morphogenesis	980	11	2.41	4.57	+	2.32E-05	3.65E-02
negative regulation of multicellular organismal process	1154	12	2.83	4.24	+	1.96E-05	3.87E-02
epithelium development	1161	12	2.85	4.21	+	2.08E-05	3.65E-02
↳tissue development	1785	16	4.38	3.65	+	3.88E-06	1.53E-02

B

GO cellular component complete	Mus musculus (REF) #	upload_1 (▽ Hierarchy NEW! ⓘ) #	expected	Fold Enrichment	+/-	raw P value	FDR
extracellular region	2875	20	7.06	2.83	+	7.91E-06	1.61E-02

Fig. S16. Gene ontology enrichment of commonly upregulated genes in female mole testis part and mouse *Sall1*-overexpressing mutant ovaries.

A. GO terms for biological processes.

B. GO terms for cellular components.

Development • Supplementary information

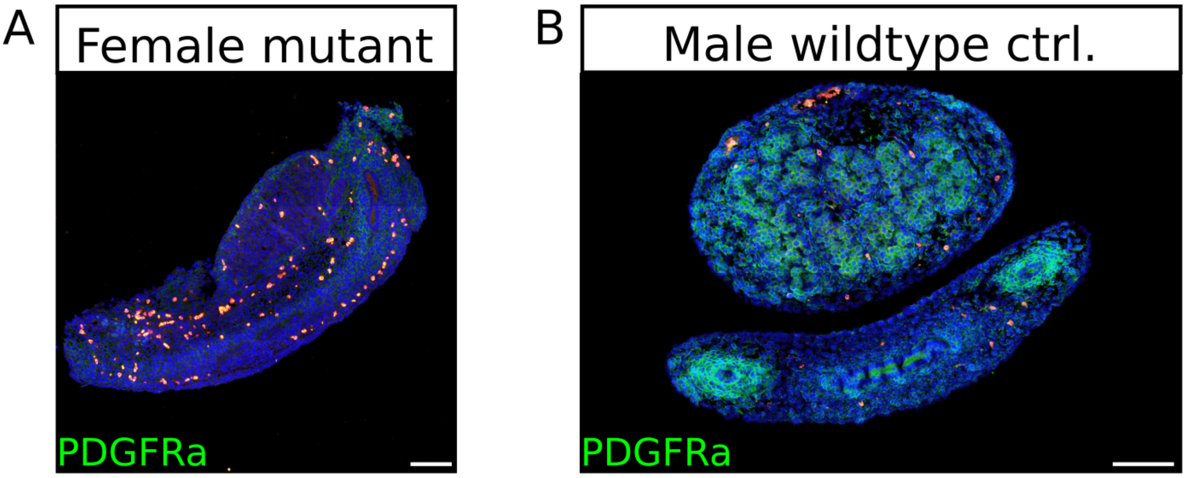

C

Gene	baseMean	log2FoldChange	lfcSE	stat	pvalue	padj
Lhx1	76,0142476	0,416247256	0,45549231	0,91384036	0,36080074	0,99999929
Fgf9	34,7898814	-0,34199249	0,64606248	-0,5293489	0,59656341	0,99999929
Cxcr4	269,917075	0,363473378	0,24191365	1,50249224	0,13297003	0,99999929
Cxcl12	867,206005	-0,400398642	0,1872609	-2,138186	0,03250165	0,99999929
Spry2	246,127611	0,017530695	0,24731929	0,07088285	0,943491	0,99999929
Mmp14	2980,353	-0,004484855	0,11824949	-0,0379271	0,96974584	0,99999929

Fig. S17. Expression of migration markers in *Sall1*-overexpressing ovaries.

A, B. Immunostaining for PDGFRa in female mutant and male wildtype controls at E13.5. There is no signal for PDGFRa in mutant ovaries, denoting the absence of migration. Scale bars: 100 µm

C. Differential gene expression between female mutant and controls for several genes involved in cell migration. Note, there are no significant differences between both conditions.

Development • Supplementary information

Table S1. Ranking of enhancer regions.

Click here to download Table S1

Table S2. Ranking of transcription factors by significance of binding affinity to the five *SALL1* enhancer sequences.

Click here to download Table S2

Table S3. Differential gene expression between *Sall1*-overexpressing mutant and wildtype ovaries.

Click here to download Table S3

Table S4. Primer list.

RT-qPCRs	
Hedgehog-qPCR-Sall1-Fwd	GAAGCAAGCGAAGCCTCAAC
Hedgehog-qPCR-Sall1-Rev	TGCTCTTAGTGGGGCGATTT
Hedgehog-qPCR-Foxl2-Fwd	CAGAAGCCGCCCTATTCGT
Hedgehog-qPCR-Foxl2-Rev	GGGAACTTGGCGATGATGT
Hedgehog-qPCR-Rps9-Fwd	GCCAAGTCCATCCACCAC
Hedgehog-qPCR-Rps9-Rev	CCAGGCGGACAATGAAGG
Shrew-qPCR-Sall1-Fwd	AGAGCGTTCACAACAAAAGG
Shrew-qPCR-Sall1-Rev	TGGGGCCATCCACAGAGA
Shrew-qPCR-Foxl2-Fwd	CATCGCCAAGTTCCCCTTCT
Shrew-qPCR-Foxl2-Rev	GCACTCGTTGAGGCTGAGGT
Shrew-qPCR-Rps9-Fwd	GAGTCCAGGCGAACAATGAA
Shrew-qPCR-Rps9-Rev	GGCCAAGTCCATCCACCA

4C-seq experiments	
Sall1-4C-Fwd	TCAGTGGGCTGACATTTTA
Sall1-4C-Rev	TCAGTGGGCTGACATTTTA
5ITR-4C-Fwd	gctgcacctacagtttggat
5ITR-4C-Rev	gctgcacctacagtttggat
3ITR-4C-Fwd	gctgcacctacagtttggat
3ITR-4C-Rev	gctgcacctacagtttggat

Amplification of the Enhancers	
Sall1-E1-Fwd	TCTGGAGAACACTCACACCC
Sall1-E1-Rev	GCAAGCCAGTAGATACCGCA
Sall1-E2-Fwd	ACTCTTTCACATGTGCCAAA
Sall1-E2-Rev	TCCAGCACAAGAAATCCTGC
Sall1-E3-Fwd	GAAAAAAAAATCTTAGGTGC
Sall1-E3-Rev	GAGCAAACAACAGCCTTCCC
Sall1-E4-Fwd	GTTTGTTCAATTTTTAAATT
Sall1-E4-Rev	ACATTGGCCTAGAAGGTATC
Sall1-E5-Fwd	CAGGGGAAGGAAGGCAGGCT
Sall1-E5-Rev	GTGGGACCCTTGCCGGTGGC

PiggyBac Wt1-Sall1-BAC Cloning	
Sall1-CDS-attB1	GGGGACAAGTTTGTACAAAAAAGCAGGCTTTTGAGCCAGCATGTCGCGG
Sall1-CDS-attB2	GGGGACCACTTTGTACAAGAAAGCTGGGTTCTGGCAGCTTTAGCTTGTG
Neo-Rec-Fwd	TGGGTAAGGCAGTGATGACAGATCAAAAGTAAAAGGTCTCACCCAGTCTACTCGACTGC ACGCGTTATATAG
Neo-Rec-Rev	TAAATAACCCCTCCTTTGTGTTCCTCTAACCCACTTAAATTTATTGCTTCATGTACCTGA CTGATGAAGTTC

Genotyping Sall1-BAC insertion into ES cells	
Sex-PCR-Fwd	CTGAAGCTTTTGGCTTTGAG
Sex-PCR-Rev	CCACTGCCAAATTCTTTGG
5'ITR-BAC-Fwd	gacgcatgcattcttgaaat
5'ITR-BAC-Rev	atgcgtcattttgactcacg
3'ITR-BAC-Fwd	gaagaaattttgagtttttgttttt
3'ITR-BAC-Rev	cgcatgtgttttatcggtct
bck-BAC-Fwd	GGCGGTGTTGATACAGCGGGTAA
bck-BAC-Rev	CCGGCGTTCGGTCGAAGAGTATC

Development • Supplementary information
