# Supplementary material for: Induction of kidney-related gene programs through co-option of SALL1 in mole ovotestes
Source: Development. 2023 Sep 5;150(17):dev201562. doi: 10.1242/dev.201562 (PMC10499028; doi:10.1242/dev.201562)
Supplement: Supplementary file 1 [file develop-150-201562_review_history.pdf]

Induction of kidney-related gene programs through co-option of SALL1 in mole ovotestes

Magdalena Schindler, Marco Osterwalder, Izabela Harabula, Lars Wittler, Athanasia C. Tzika, Dina K.N. Dechmann, Martin Vingron, Axel Visel, Stefan A. Haas and Francisca M. Real
DOI: 10.1242/dev.201562

Editor: Cassandra Extavour

Review timeline

Original submission:	30 December 2022
Editorial decision:	16 February 2023
First revision received:	2 June 2023
Accepted:	21 July 2023

Original submission

First decision letter

MS ID#: DEVELOP/2022/201562

MS TITLE: Co-option of the transcription factor SALL1 in mole ovotestis formation

AUTHORS: Magdalena Schindler, Marco Osterwalder, Izabela Harabula, Lars Wittler, Athanasia C. Tzika, Dina K.N. Dechmann, Martin Vingron, Axel Visel, Stefan A. Haas, and Francisca M. Real

I have now received all the referees' reports on the above manuscript, and have reached a decision. The referees' comments are appended below, or you can access them online: please go to BenchPress and click on the 'Manuscripts with Decisions' queue in the Author Area.

As you will see, the referees express considerable interest in your work, but have some significant criticisms and recommend a substantial revision of your manuscript before we can consider publication. If you are able to revise the manuscript along the lines suggested, which may involve further experiments, I will be happy receive a revised version of the manuscript. Your revised paper will be re-reviewed by one or more of the original referees, and acceptance of your manuscript will depend on your addressing satisfactorily the reviewers' major concerns. Please also note that Development will normally permit only one round of major revision. If it would be helpful, you are welcome to contact us to discuss your revision in greater detail. Please send us a point-by-point response indicating your plans for addressing the referee’s comments, and we will look over this and provide further guidance.

Please attend to all of the reviewers' comments and ensure that you clearly highlight all changes made in the revised manuscript. Please avoid using 'Tracked changes' in Word files as these are lost in PDF conversion. I should be grateful if you would also provide a point-by-point response detailing how you have dealt with the points raised by the reviewers in the 'Response to Reviewers' box. If you do not agree with any of their criticisms or suggestions please explain clearly why this is so.

© 2023. Published by The Company of Biologists under the terms of the Creative Commons Attribution License (https://creativecommons.org/licenses/by/4.0/).

Reviewer 1

Advance summary and potential significance to field

This study aims at understanding the role of cis-regulatory changes in the evolution of an organ, namely ovaries. They take advantage of female Talpid moles that develop ovotestes (fertile ovaries that comprise a sterile testicular portion). These modified organs increase muscle strength and aggression –a possible adaptation to subterranean life.

Authors previously showed that ovotestes development is mediated by TAD reorganization: a large chromosomal inversion produces ectopic FGF9 expression and a duplication of enhancers increases CYP17A1 expression. Here, using various genomic and epigenetic analyses, they characterize and compare all regulatory elements in mole and mouse gonads, and observe that only 1/4th of enhancers is active in both species. This suggests a repurposing of enhancer function during gonad evolution.

Among differentially expressed genes, they chose to focus on SALL1, which a transcription regulator involved in cell fate decision that strongly and specifically marks the testicular part of ovotestes. Using immunostaining, they find that contrary to moles, SALL1 is not expressed in the female gonads of mice, hedgehogs and common shrews, tough its locus, part of large TAD, is largely conserved between species.

By overlaying mouse/mole epigenetic data with TADs analyses, they identify 5 enhancer elements putatively interacting with the SALL1 promoter in moles (but not in mice), that they test in mouse transgenic lacZ reporter assays. None display specific gonad expression, but 4 are active in the mesonephros, which has the same ontogenic origin as ovotestes.

Finally, authors present results obtained by producing transgenic mice ectopically expressing SALL1 in ovaries. They find this has no effect on gonad formation and fertility, but de-regulates 400 genes, part of those being also specifically expressed in mole ovotestis. Together, data suggest that SALL1 has been co-opted in mole ovotestes.

Comments for the author

This is a clear, to-the-point, well-written manuscript. Results have strong significance, because they address long-standing problems (i.e. the impact and mechanisms of gene co-option in the evolution of an adaptive phenotype) using a very original model (mole ovotestes) and through an elegant combination of genomic work, expression analyses, and functional assays –the latter being very rarely undertaken in higher vertebrates. I however have the following concerns:

- Functional tests have been performed to validate the role of SALL1 in mole ovotestes, this is a rare and understandably difficult undertaking; however, results remain somewhat of a drawback, as they do not fully provide evidence for co-option of SALL1 in mole ovotestes (TADs are conserved, Lacz experiments do not show enhancer activity in testes, and ectopic expression in mice does not modify tissue structure and reproductive abilities). With this is mind, I find that authors' claims on the role of SALL1 in moles are too strong and definitive. Further functional tests should be performed in moles (perhaps using Crispr-Cas9 technology, otherwise used in many non-model organisms?). Alternatively, and considering this would diminish the impact of the study, the overall writing should tune-down causative claims.

- In light of the absence of direct evidence for a role of SALL1 in ovotestes, authors argue that SALL1 enhancers have likely been co-opted as some are active in the mesonephros, which has the same ontogenic origin as ovotestes.
It would thus be informative (and strengthening authors claims) to compare SALL1 expression in moles and mice/hedgehogs/shrews and/or test the effect of SALL1 mis-expression in the mesonephros (i.e. in transgenic mice)

- Why has the ovarian marker FOXL2 not been used in moles? This would be important for data robustness and consistency

© 2023. Published by The Company of Biologists under the terms of the Creative Commons Attribution License (https://creativecommons.org/licenses/by/4.0/).

- A mouse-specific antibody is used to detect SALL1 in all species. Given commonly-encountered technical problems while using antibodies in non-model species, an absence of expression (such as observed in hedgehogs and shrews)

Could not be shown using immunocytochemistry alone. What about qPCR?

Minor comments:
- Please define “RKPM”
- Line 328: the word “demonstrate” is too strong. Please modify (as well as in other similar sentences in the manuscript).
- Sall1 is sometimes capitalized (SALL1) , sometimes not (Sall1), please homogenize or specify.

Reviewer 2

Advance summary and potential significance to field

This group previously reported the mole genome sequence and molecular mechanisms underlying the development of ovotestis. Here they extended the study to investigation of a transcription factor, SALL1, which was mostly expressed in TP of ovotestis. They found five mole-specific enhancers for SALL1 in an evolutionary conserved TAD and tested the activity in TG mice. As a result, the enhancers could activate the transcription in urogenital tissues while gonadal expression of a reporter gene was not observed. Overexpression of SALL1 in the mouse ovary induced transcriptional activation of genes related to kidney development. They concluded the co-option of gene expression through changes in enhancer activity. The study provides a precious example of the co-option of gene regulation in mole.

Comments for the author

[Major points]
1) Because the co-option of gene regulation has been reported elsewhere in other species, the significance of the current finding is not necessarily clear. The authors should mention what is known about the co-option of gene expression/regulation in the introduction and discuss the significance of this study in the discussion.

2) Immunostaining of SALL1 is important in this manuscript. The authors could not detect any positive signals in hedgehog and shrew, but the antibody they used (ab41974) is described to only react with mouse antigen. This indicates the possibility that the antibody simply could not detect the protein in hedgehog and shrew. The usage of this antibody should be validated. If SALL1 is expressed in hedgehog and/or shrew, the interpretation may be changed. They may also check the expression of SALL1 mRNA in these species if the data are available.

[Minor comments]
-Lines 84-86: Please add a reference to this sentence.
-Lines132-133: The SALL1 expression was also detected in OP. “specific to the testis part” is a bit overstatement.
-Lines 154-155: From the provided figure, it is hard to tell “restricted to the Sertoli-like cell”. Is there additional evidence supporting this conclusion?
-Lines 474-478: No authors belong to University of Granada. Who provided the mole for this study?

Reviewer 3

Advance summary and potential significance to field

Schindler et al. seek to identify novel regulators of mole ovotestis development. By comparing ChIP-seq datasets produced in mole gonads to analogous datasets in the mouse, they identify a set

© 2023. Published by The Company of Biologists under the terms of the Creative Commons Attribution License (https://creativecommons.org/licenses/by/4.0/).

of mole-specific enhancers active specifically in the testicular part of the ovotestis. Combining enhancer score and specificity of expression of the associated gene, they identify SALL1 as one of the top ranked target genes in the testis part of the ovotestis. Using transcriptomic analysis and immunohistochemistry, the authors confirm SALL1 as a marker for the testis part. They identify five regulatory elements in contact with the SALL1 promoter which are active in specifically in the testis part of the mole ovotestis. Next, the authors test the activity of these five mole elements in vivo using LacZ reporter assays in mice and find that while none of the elements drive LacZ expression in the gonads, 4 out of the 5 are active in the mesonephros. Lastly, they overexpress SALL1 in developing mouse ovaries and find that 92 differentially expressed genes in mutant gonads which are also enriched in the testis part of the ovotestis.

The authors set out to address a very interesting question, and despite the fact that many results are negative, the study is sound and the methodology is appropriate. I have a few comments and suggestions that could improve the manuscript and strengthen the evidence of the claims.

Comments for the author

1. The authors discuss the idea that enhancers active in the mouse mesonephros are co-opted in the ovotestis, and that migratory cells from the mesonephros may contribute to the formation of the ovotestis. While this idea is intriguing, it is underdeveloped and the evidence presented is weak. It is known that cell migration from the mesonephros is an important initiating step in testes development. Can the authors show that such migration occurs in the mole ovotestis, perhaps conferring a testicular identity within the developing ovaries? A deeper investigation of the existence of such a migration effect is needed. This has been done in mouse using in vitro recombination experiments (doi:10.1016/s0925-4773(99)00047-7). I realize this is a tool that is difficult to implement in moles but can the authors perform histological analysis and/or immunohistochemistry of the developing gonad?

2. The authors state that mole enhancers were not able to recapitulate gonadal expression in mouse reporter assays possibly due to additional trans-acting factors which may be required for their activation. Are there putative transcription factor binding motifs enriched within enhancers active specifically in the testicular part of the ovotestis? Moreover, alignment of the five regulatory elements in contact with the SALL1 promoter against mouse and shew revealed only a partial degree of sequence conservation. Are there potential transcription factor binding motifs present in mole-specific segments of the five regulatory elements tested that may explain changes in regulatory activity due to cis regulatory differences?

3. While SALL1 overexpression in the mouse gonads was found to be insufficient to induce the development of testicular structures in the ovary, the effect of SALL1 expression on testicular development in males was not reported. Does SALL1 overexpression result in any morphological testicular alterations in male mice?

Moreover, is there any effect on migration of cells from the mesonephros upon SALL1 overexpression?

4. The authors show using immunostaining that SALL1 is absent from the ovaries of other species from the order Eulipotyphla, including Atelerix albiventris and Sorex araneus. For this experiment, positive controls are needed showing this antibody is functional in these species. This could be accomplished by performing immunostaining for SALL1 in the kidney of these two species.

5.“This observation, together with the moderate sequence conservation compared to other mammals suggests that the evolution of adjacent enhancers drives SALL1 expression in the testicular part of mole ovotestis." (lines 224-226)

It is unclear exactly what is meant by this statement. Are the authors referring to ‘adjacent enhancers’ as in enhancers which are active in an adjacent tissue (the mesonephros), or to regions within the enhancer candidates which don’t share sequence homology? Please clarify

© 2023. Published by The Company of Biologists under the terms of the Creative Commons Attribution License (https://creativecommons.org/licenses/by/4.0/).

6. "Our results highlight the co-option of gene expression, through changes in enhancer activity, as a prominent mechanism for the evolution of traits." (lines 34-35)
"In contrast, our current study also highlight that the evolution of CREs within conserved TADs is a prominent mechanism for evolution." (lines 323-324)

This language seems misleading in that it suggests that the co-option of SALL1 has played a causative role in the emergence of ovotestis in moles. While the authors have shown that SALL1 is a marker for the testis part of the mole ovotestis, the experiments performed have not shown the requirement nor the sufficiency of SALL1 for ovotestis development. Given the evidence presented, it appears it remains an open question whether the co-option of SALL1 expression may have been involved in the evolution of this novel trait. In general, the language used in the manuscript and the title should be modified to match what the data shows.

First revision

Author response to reviewers' comments

Reviewer 1 Advance Summary and Potential Significance to Field:
This study aims at understanding the role of cis-regulatory changes in the evolution of an organ, namely ovaries. They take advantage of female Talpid moles that develop ovotestes (fertile ovaries that comprise a sterile testicular portion). These modified organs increase muscle strength and aggression –a possible adaptation to subterranean life. Authors previously showed that ovotestes development is mediated by TAD reorganization: a large chromosomal inversion produces ectopic FGF9 expression, and a duplication of enhancers increases CYP17A1 expression. Here, using various genomic and epigenetic analyses, they characterize and compare all regulatory elements in mole and mouse gonads, and observe that only 1/4th of enhancers is active in both species. This suggests a repurposing of enhancer function during gonad evolution. Among differentially expressed genes, they chose to focus on SALL1, which a transcription regulator involved in cell fate decision that strongly and specifically marks the testicular part of ovotestes. Using immunostaining, they find that contrary to moles, SALL1 is not expressed in the female gonads of mice, hedgehogs and common shrews, tough its locus, part of large TAD, is largely conserved between species. By overlaying mouse/mole epigenetic data with TADs analyses, they identify 5 enhancer elements putatively interacting with the SALL1 promoter in moles (but not in mice), that they test in mouse transgenic lacZ reporter assays. None display specific gonad expression, but 4 are active in the mesonephros, which has the same ontogenic origin as ovotestes. Finally, authors present results obtained by producing transgenic mice ectopically expressing SALL1 in ovaries. They find this has no effect on gonad formation and fertility, but de-regulates 400 genes, part of those being also specifically expressed in mole ovotestis. Together, data suggest that SALL1 has been co-opted in mole ovotestes.

Reviewer 1 Comments for the Author:
This is a clear, to-the-point, well-written manuscript. Results have strong significance, because they address long-standing problems (i.e. the impact and mechanisms of gene co-option in the evolution of an adaptive phenotype) using a very original model (mole ovotestes) and through an elegant combination of genomic work, expression analyses, and functional assays –the latter being very rarely undertaken in higher vertebrates. I however have the following concerns:

-Functional tests have been performed to validate the role of SALL1 in mole ovotestes, this is a rare and understandably difficult undertaking; however, results remain somewhat of a drawback, as they do not fully provide evidence for co-option of SALL1 in mole ovotestes (TADs are conserved, Lacz experiments do not show enhancer activity in testes, and ectopic expression in mice does not modify tissue structure and reproductive abilities). With this is mind, I find that authors' claims on the role of SALL1 in moles are too strong and definitive. Further functional tests should be performed in moles (perhaps using Crispr-Cas9 technology, otherwise used in many non-model organisms?). Alternatively, and considering this would diminish the impact of the study, the overall writing should tune-down causative claims.

© 2023. Published by The Company of Biologists under the terms of the Creative Commons Attribution License (https://creativecommons.org/licenses/by/4.0/).

We would like to thank the reviewer for recognizing the challenges of working with non-classical model organisms. We agree that genetically modifying moles would be the ideal approach to prove the role of *SALL1* in ovotestis development. Unfortunately, such experiments are not feasible as moles cannot be maintained in captivity. In the past, we and others have made remarkable efforts in establishing moles colonies, but reality is that they generally do not survive in captivity, nor are they able to reproduce. Therefore, we rely on intensive field work to capture wild specimens using a trapping system that does not harm the animals. To perform functional validations, we use transgenic mice as a useful model to determine the impact of mole-specific regulatory changes, as we have shown previously (Real *et al.,* Science 2020). On the one hand, we understand that cross-species experiments have their own caveats and limitations. But, on the other hand, they provide an alternative to validate findings from studies performed in non-classical model organisms.

As we could not provide a complete proof for the involvement of *SALL1* expression in ovotestes development (which would require the generation of *SALL1*-knockout moles), we have toned down the strength of our statements throughout the entire manuscript, following the reviewer's advice. The title has been also modified to provide a more accurate description of the main findings of the study. Even in the absence of transgenic moles, we were able to demonstrate that *SALL1* is sufficient to induce kidney-related gene programs that are also observed during mole ovotestes development. Further, we identified enhancers exhibiting tissue-restricted activity in transgenic reporter mice and a distinct biochemical signature that is specific to the testicular part of the ovotestes. In this same tissue we identified an "ectopic" expression of *SALL1*, which constitutes a bona-fide marker of the testicular part of the ovotestes. Although *Sall1*-overexpressing mice did not display obvious morphological phenotypic changes, they did show a molecular phenotype with similarities to ovotestes development. We believe that this revised version better acknowledges the limitations of our study, avoids overinterpretation of the results, while still recognizing the significance of our findings.

All changes in the manuscript are highlighted through track changes.

-In light of the absence of direct evidence for a role of SALL1 in ovotestes, authors argue that SALL1 enhancers have likely been co-opted as some are active in the mesonephros, which has the same ontogenic origin as ovotestes. It would thus be informative (and strengthening authors claims) to compare SALL1 expression in moles and mice/hedgehogs/shrews and/or test the effect of SALL1 mis-expression in the mesonephros (i.e. in transgenic mice)

We performed immunostaining to examine the expression of SALL1 in the mesonephros of moles and mice, as we did not have access to embryonic gonads of shrews and hedgehogs. We observed a consistent pattern of expression in both species, suggesting that this pattern is likely conserved in other related species. Specifically, we found that SALL1 was expressed in the mesonephric duct, consistent with a previous publication reporting similar findings (Nishinakamura *et al.*, Development 2001). This is an interesting observation that aligns with the results from our enhancer reporter assays. In these transgenic mice, we detected activity for the mole enhancers in the same tissue where SALL1 is expressed, consistent with our hypothesis that similar trans-acting factors that activate SALL1 in the mouse mesonephros could be expressed in the female ovotestis. We are grateful to the reviewer for bringing this to our attention and have accordingly added a new supplementary figure and text to the manuscript to further support this finding.

Regarding the effects of a misexpression on the mesonephros, SALL1 is already expressed in this tissue during mouse development. Therefore, the mesonephric expression of SALL1 does not influence the fate of the gonad.

-*Why has the ovarian marker FOXL2 not been used in moles? This would be important for data robustness and consistency*

To confirm that the tissues were processed correctly, as there was no signal for SALL1, we used the FOXL2 marker as a positive control for immunostainings in hedgehog and shrew gonads. Following the reviewer´s advice, we have now included a double staining image of SALL1/FOXL2 in moles in Figure 2 for consistency. This control staining also demonstrates the specificity of SALL1 to the testicular portion, as there were no double positive cells in the ovarian part.

© 2023. Published by The Company of Biologists under the terms of the Creative Commons Attribution License (https://creativecommons.org/licenses/by/4.0/).

-A mouse-specific antibody is used to detect SALL1 in all species. Given commonly-encountered technical problems while using antibodies in non-model species, an absence of expression (such as observed in hedgehogs and shrews) could not be shown using immunocytochemistry alone. What about qPCR?

All reviewers raised this important concern that we have addressed in two ways. Firstly, we have performed immunostainings for SALL1 in control tissues from hedgehogs and shrews to confirm the specificity of the antibody. Our results showed that the antibody is able to recognize the SALL1 protein in these species, as evidenced by specific expression in the neural tube of hedgehog embryos and in the adult kidneys of shrew, both of which are well-known SALL1 expressing tissues.

Secondly, we have performed RT-qPCRs for *SALL1* and *FOXL2* in adult ovaries and kidneys from hedgehogs and shrews. The results corroborated our immunostainings findings, with no expression of *SALL1* in the ovaries but high expression of *FOXL2*, and high expression of *SALL1* in the kidneys of both species.

We have included a new supplementary figure and update the text accordingly.

Minor comments:
-Please define "RKPM"
-Line 328: the word "demonstrate" is too strong. Please modify (as well as in other similar sentences in the manuscript).
-Sall1 is sometimes capitalized (SALL1) , sometimes not (Sall1), please homogenize or specify.

- "RPKM" is reads per kilobase million and is defined now in the text.
- Line 328: demonstrate has been substituted for "suggest". Similar changes have been also made throughout the manuscript (reflected by the track changes).
- We have made an effort to adhere to the standard guidelines for nomenclature of genes and proteins, which typically involves italicizing gene names and writing them in non-capitalized letters for mouse and capitalized for human and other non-rodent species such as moles. Protein names are always capitalized and not italicized. To provide consistency, we have reviewed and revised the entire manuscript to ensure that all gene and protein names are written in the appropriate form.

Reviewer 2 Advance Summary and Potential Significance to Field:
This group previously reported the mole genome sequence and molecular mechanisms underlying the development of ovotestis. Here they extended the study to investigation of a transcription factor, SALL1, which was mostly expressed in TP of ovotestis. They found five mole-specific enhancers for SALL1 in an evolutionary conserved TAD and tested the activity in TG mice. As a result, the enhancers could activate the transcription in urogenital tissues while gonadal expression of a reporter gene was not observed. Overexpression of SALL1 in the mouse ovary induced transcriptional activation of genes related to kidney development. They concluded the co-option of gene expression through changes in enhancer activity. The study provides a precious example of the co-option of gene regulation in mole.

Reviewer 2 Comments for the Author:
[Major points]

1) Because the co-option of gene regulation has been reported elsewhere in other species, the significance of the current finding is not necessarily clear. The authors should mention what is known about the co-option of gene expression/regulation in the introduction and discuss the significance of this study in the discussion.

We apologize for not introducing the concept of co-option in the introduction. To address this issue, we have added a new paragraph that includes relevant citations and examples. As we show in our study, the gene *SALL1*, primarily known for its role in kidney development, has acquired expression in the mole ovotestis. This novel expression in gonadal tissue can trigger the expression

© 2023. Published by The Company of Biologists under the terms of the Creative Commons Attribution License (https://creativecommons.org/licenses/by/4.0/).

of kidney- related genes, as denoted in our mouse overexpression model and our expression analyses in mole ovotestes. The distinct expression of *SALL1* in mole ovotestes is associated with the activation of several enhancers, which are also capable of activating transcription in other SALL1-expressing tissues. As such, our study further extends our understanding of the origin of this unique trait and, at the same time, provides an example on how changes in regulation can modify gene expression programs, as indicated in the discussion.

All modifications in the text are reflected by the track changes.

2) Immunostaining of SALL1 is important in this manuscript. The authors could not detect any positive signals in hedgehog and shrew, but the antibody they used (ab41974) is described to only react with mouse antigen. This indicates the possibility that the antibody simply could not detect the protein in hedgehog and shrew. The usage of this antibody should be validated. If SALL1 is expressed in hedgehog and/or shrew, the interpretation may be changed. They may also checkthe expression of SALL1 mRNA in these species if the data are available.

All the reviewers raised this concern that we have addressed in two ways. Firstly, we have performed immunostainings for SALL1 in control tissues from hedgehogs and shrews to confirm the specificity of the antibody. Our results showed that the antibody recognizes SALL1 protein perfectly in these species, as evidenced by specific expression in the neural tube of hedgehog embryos and in the adult kidneys of shrew, both of which are well-known SALL1 expressing tissues.

Secondly, we have performed RT-qPCRs for *SALL1* and *FOXL2* in adult ovaries and kidneys from hedgehogs and shrews. The results corroborated our immunostainings findings, with no expression of *SALL1* in the ovaries but high expression of *FOXL2*, and high expression of *SALL1* in the kidneys of both species.

We have included a new supplementary figure and update the text accordingly.

[Minor comments]
-Lines 84-86: Please add a reference to this sentence.
-Lines132-133: The SALL1 expression was also detected in OP. “specific to the testis part” is a bit overstatement.
-Lines 154-155: From the provided figure, it is hard to tell “restricted to the Sertoli-like cell”. Is there additional evidence supporting this conclusion?
-Lines 474-478: No authors belong to University of Granada. Who provided the mole for this study?

- Lines 84-86: References have been added.
- Lines 132-133: Our previous claim that SALL1 expression is specific to the testis part was based on immunostainings, which did not show any expression in the ovarian part. To further support this, we have included a double immunostaining image for SALL1 and FOXL2 in Figure 2, which shows that double positive cells are only present in the testicular part, while the ovarian part is positive only for FOXL2. However, we acknowledge that the RNA sequencing plots indicate some *SALL1* expression in the ovary, particularly in the adult gonad. This is likely due to the difficulty of completely separating the two parts during dissection, especially in the adult organ where they are more connected. To address this, we have added a sentence in the text to clarify the issue with microdissection.
- Lines 154-155: As mentioned, we have added a double immunostaining for FOXL2/SALL1 to further support our findings. The staining shows that the cells that are double positive for FOXL2 and SALL1 form the spherules, which are the equivalent of testis cords, and are therefore considered "Sertoli- like" cells.
- Lines 474-478: as reflected in several of our publications, we have had a longstanding collaboration with the lab of Prof. Jiménez at the University of Granada, who kindly hosted us to obtain the samples. In addition, most of the mole material for this study was reanalyzed from previously published studies. To clarify this, we have added Prof. Jiménez's name to the permission for capturing moles and thanked him for his assistance in the acknowledgements section.

© 2023. Published by The Company of Biologists under the terms of the Creative Commons Attribution License (https://creativecommons.org/licenses/by/4.0/).

Reviewer 3 Advance Summary and Potential Significance to Field:
Schindler et al. seek to identify novel regulators of mole ovotestis development. By comparing ChIP- seq datasets produced in mole gonads to analogous datasets in the mouse, they identify a set of mole- specific enhancers active specifically in the testicular part of the ovotestis. Combining enhancer score and specificity of expression of the associated gene, they identify SALL1 as one of the top ranked target genes in the testis part of the ovotestis. Using transcriptomic analysis and immunohistochemistry, the authors confirm SALL1 as a marker for the testis part. They identify five regulatory elements in contact with the SALL1 promoter which are active in specifically in the testis part of the mole ovotestis. Next, the authors test the activity of these five mole elements in vivo using LacZ reporter assays in mice and find that while none of the elements drive LacZ expression in the gonads, 4 out of the 5 are active in the mesonephros. Lastly, they overexpress SALL1 in developing mouse ovaries and find that 92 differentially expressed genes in mutant gonads which are also enriched in the testis part of the ovotestis.

The authors set out to address a very interesting question, and despite the fact that many results are negative, the study is sound and the methodology is appropriate. I have a few comments and suggestions that could improve the manuscript and strengthen the evidence of the claims.

***Reviewer 3 Comments for the Author*:**

1. The authors discuss the idea that enhancers active in the mouse mesonephros are co-opted in the ovotestis, and that migratory cells from the mesonephros may contribute to the formation of the ovotestis. While this idea is intriguing, it is underdeveloped and the evidence presented is weak. It is known that cell migration from the mesonephros is an important initiating step in testes development. Can the authors show that such migration occurs in the mole ovotestis, perhaps conferring a testicular identity within the developing ovaries? A deeper investigation of the existence of such a migration effect is needed. This has been done in mouse using in vitro recombination experiments (doi:10.1016/s0925-4773(99)00047-7). I realize this is a tool that is difficult to implement in moles but can the authors perform histological analysis and/or immunohistochemistry of the developing gonad?

This an important point that we wondered about and we also investigated it in the past. However, the methodology described in that article is unfeasible to apply to moles as the capture of pregnant females cannot be programmed. To implement this experiment in moles, it would involve having GFP female mouse plugs every day for several months to coordinate the capture of a mole pregnant female at the same stage as the pregnant mice. But a few years ago, we obtained indirect evidences of migration in the developing female mole gonad (Carmona *et al.*, J Exp Zool B Mol Dev Evol., 2009). In this study, we performed immunostainings against a couple of migration markers (PGDRFa and MT1- MMP) and observed that the developing ovotestis exhibits a similar pattern to that of the male testis, in contrast to what is observed in mice, where migration is only present in the male gonad. At the time, it was hypothesized that these indirect signs of migration in the female gonad could contribute to the formation of the testicular tissue in the ovotestis. Based on our current data, which show the presence of similar genetic programs in both the mesonephros and ovotestis that can be activated by *SALL1*, this hypothesis remains plausible.

We have now included the previous findings of the migration markers earlier in the manuscript to facilitate the reader's understanding. The modifications made to the text are highlighted with the track changes.

2. The authors state that mole enhancers were not able to recapitulate gonadal expression in mouse reporter assays possibly due to additional trans-acting factors which may be required for their activation. Are there putative transcription factor binding motifs enriched within enhancers active specifically in the testicular part of the ovotestis? Moreover, alignment of the five regulatory elements in contact with the SALL1 promoter against mouse and shew revealed only a partial degree of sequence conservation. Are there potential transcription factor binding motifs present in mole-specific segments of the five regulatory elements tested that may explain changes in regulatory activity due to cis regulatory differences?

The questions raised by the reviewer were very intriguing, so we conducted an enrichment analysis for transcription factor binding motifs in the mole enhancers and compared them to the sequences

© 2023. Published by The Company of Biologists under the terms of the Creative Commons Attribution License (https://creativecommons.org/licenses/by/4.0/).

of shrew and mouse. We then filtered these motifs based on the specific expression of putative transcription factors in the mole gonadal tissues. As pointed out by the reviewer, there are significant differences in transcription factor binding among species due to partial conservation between them. For instance, if we consider the top 50 TF binding motifs for each species, only 8 and 9 (16% and 18%) are shared between mole and mouse and shrew, respectively. Moreover, there are significant differences in the expression levels of certain top-ranked TF between the mole and mouse gonads. This observation suggests that variations in TF binding and expression within divergent enhancer sequences may contribute to the specific activation of *SALL1* in mole ovotestes, while providing a possible explanation of the absence of lacZ expression in the transgenic mouse gonads.

We have added a new supplementary table containing these results and provided an explanation in the text. We would like to express our gratitude to the reviewer for this suggestion, which has further supported our main findings.

3. While SALL1 overexpression in the mouse gonads was found to be insufficient to induce the development of testicular structures in the ovary, the effect of SALL1 expression on testicular development in males was not reported. Does SALL1 overexpression result in any morphological testicular alterations in male mice? Moreover, is there any effect on migration of cells from the mesonephros upon SALL1 overexpression?

We have reported now the status of the male gonad development upon *Sall1* overexpression. Hematoxylin-eosin staining was performed on mutant testes and controls at both prenatal and postnatal stages. However, no morphological differences were observed between the mutant and wildtype testes. Furthermore, these mutant males were able to breed normally and produce litters in normal Mendelian ratios, indicating that neither gonad development nor fertility was affected by *Sall1* overexpression. To report these findings, we have added a supplementary figure and revised the text accordingly.

We investigated the status of migration in female mutant gonads using two approaches. First, we performed immunostainings against PDGFRa, a migration marker expressed solely in testes in mice but in testes and ovotestes in moles, which was negative in our *Sall1*-overexpressing ovaries. Second, we compared the expression values of several migration markers in the RNA-seq data of mutants and control gonads. However, none of these genes were significantly deregulated in the mutant versus control ovaries. Our results suggest that no migration occurs in these mutant ovaries, which may explain why we did not observe morphological changes resembling the formation of testicular tissue. We added a new supplementary figure to illustrate these findings and discussed them in the text.

4. The authors show using immunostaining that SALL1 is absent from the ovaries of other species from the order Eulipotyphla, including Atelerix albiventris and Sorex araneus. For this experiment, positive controls are needed showing this antibody is functional in these species. This could be accomplished by performing immunostaining for SALL1 in the kidney of these two species.

All the reviewers raised this concern that we have addressed in two ways. Firstly, we have performed immunostainings for SALL1 in control tissues from hedgehogs and shrews to confirm the specificity of the antibody. Our results showed that the antibody recognizes SALL1 protein in these species, as evidenced by specific expression in the neural tube of hedgehog embryos and in the adult kidneys of shrew, both of which are well-known SALL1 expressing tissues.

Secondly, we have performed RT-qPCRs for *SALL1* and *FOXL2* in adult ovaries and kidneys from hedgehogs and shrews. The results corroborated our immunostainings findings, with no expression of SALL1 in the ovaries but high expression of FOXL2, and high expression of SALL1 in the kidneys of both species.

We have included a new supplementary figure and update the text accordingly.

5.“This observation, together with the moderate sequence conservation compared to other mammals suggests that the evolution of adjacent enhancers drives SALL1 expression in the

© 2023. Published by The Company of Biologists under the terms of the Creative Commons Attribution License (https://creativecommons.org/licenses/by/4.0/).

testicular part of mole ovotestis." (lines 224-226)

It is unclear exactly what is meant by this statement. Are the authors referring to 'adjacent enhancers' as in enhancers which are active in an adjacent tissue (the mesonephros), or to regions within the enhancer candidates which don't share sequence homology? Please clarify

We apologize for any confusion caused by our use of the word "adjacent." We meant that the regions we studied were located in the regulatory domain of the *SALL1* gene in the genome. We have revised the text to make this clearer.

6. "Our results highlight the co-option of gene expression, through changes in enhancer activity, as a prominent mechanism for the evolution of traits." (lines 34-35)

"In contrast, our current study also highlight that the evolution of CREs within conserved TADs is a prominent mechanism for evolution." (lines 323-324)

This language seems misleading in that it suggests that the co-option of SALL1 has played a causative role in the emergence of ovotestis in moles. While the authors have shown that SALL1 is a marker for the testis part of the mole ovotestis, the experiments performed have not shown the requirement nor the sufficiency of SALL1 for ovotestis development. Given the evidence presented, it appears it remains an open question whether the co-option of SALL1 expression may have been involved in the evolution of this novel trait. In general, the language used in the manuscript and the title should be modified to match what the data shows.

We appreciate the thoughtful review and acknowledge the valid concerns raised regarding the direct role of *SALL1* in the evolution of ovotestes in moles. We agree that the question of whether the co- option of *SALL1* directly caused the formation of ovotestes remains open, and definitively answering it would require generating *SALL1* knockout moles, an unfeasible experiment in this species. Therefore, we have made modifications to the title and the text throughout the entire manuscript to better align with our data-supported findings.

Despite the absence of transgenic moles, our experiments demonstrate that *Sall1* is capable of activate genetic programs that are shared during the development of ovotestes, including kidney- related programs. Moreover, we have shown a species-specific expression pattern of *SALL1* acquired in mole ovotestes, establishing *SALL1* as a definitive marker for the testicular tissue of the ovotestes. This specific expression pattern is associated with the evolution of an enhancer cluster that can activate transcription in other SALL1-expressing tissues.

While *Sall1*-overexpressing mice did not show any discernible morphological phenotype, we observed a distinct molecular phenotype with similarities to the development of ovotestes. By acknowledging the limitations of our study and avoiding misleading claims, we believe that this revised version better represents our findings and their implications without diminishing their significance.

Second decision letter

MS ID#: DEVELOP/2022/201562

MS TITLE: Induction of kidney-related gene programs through co-option of SALL1 in mole ovotestes

AUTHORS: Magdalena Schindler, Marco Osterwalder, Izabela Harabula, Lars Wittler, Athanasia C. Tzika, Dina K.N. Dechmann, Martin Vingron, Axel Visel, Stefan A. Haas, and Francisca M. Real
ARTICLE TYPE: Research Article

First, please accept my sincere apologies for the unacceptable length of time it has taken for me to gather reviews, make a decision, and relay it to you. This was due to a combination of later

© 2023. Published by The Company of Biologists under the terms of the Creative Commons Attribution License (https://creativecommons.org/licenses/by/4.0/).

reviewer response and to my own schedule, but it is not an acceptable time frame for authors like yourself who submit their work to Development, and for that I apologise.

I am happy to tell you that your manuscript has been accepted for publication in Development, pending our standard ethics checks.

Reviewer 1

Advance summary and potential significance to field

A number of additional experiments has been performed, including crucial RT-PCR tests, that confirm previous results, and strengthen conclusions. Wording has also been satisfactorily modified.

Comments for the author

Authors have addressed all my comments in a satisfactory manner.

Reviewer 2

Advance summary and potential significance to field

As I commented in the first round of review, this study provides a precious example of the co-option of gene regulation in mole.

Comments for the author

I have no further comments. The manuscript is adequately improved.

Reviewer 3

Advance summary and potential significance to field

Schindler et al. seek to identify novel regulators of mole ovotestis development. By comparing ChIPseq datasets produced in mole gonads to analogous datasets in the mouse, they identify a set of mole specific enhancers active specifically in the testicular part of the ovotestis. Combining enhancer score and specificity of expression of the associated gene, they identify SALL1 as one of the top ranked target genes in the testis part of the ovotestis. Using transcriptomic analysis and immunohistochemistry, the authors confirm SALL1 as a marker for the testis part. They identify five regulatory elements in contact with the SALL1 promoter which are active in specifically in the testis part of the mole ovotestis. Next, the authors test the activity of these five mole elements in vivo using LacZ reporter assays in mice and find that while none of the elements drive LacZ expression in the gonads, 4 out of the 5 are active in the mesonephros. Lastly, they overexpress SALL1 in developing mouse ovaries and find that 92 differentially expressed genes in mutant gonads which are also enriched in the testis part of the ovotestis.

Comments for the author

The authors had done a good job addressing my comments and the paper has improved considerably. I don't have any further reservations.

© 2023. Published by The Company of Biologists under the terms of the Creative Commons Attribution License (https://creativecommons.org/licenses/by/4.0/).
